# Mitochondrial uncoupling protein‐2 reprograms metabolism to induce oxidative stress and myofibroblast senescence in age‐associated lung fibrosis

**DOI:** 10.1111/acel.13674

**Published:** 2022-08-07

**Authors:** Sunad Rangarajan, Morgan L. Locy, Diptiman Chanda, Ashish Kurundkar, Deepali Kurundkar, Jennifer L. Larson‐Casey, Pilar Londono, Rushita A. Bagchi, Brian Deskin, Hanan Elajaili, Eva S. Nozik, Jessy S. Deshane, Jaroslaw W. Zmijewski, Oliver Eickelberg, Victor J. Thannickal

**Affiliations:** ^1^ Division of Pulmonary Sciences and Critical Care, Department of Medicine University of Colorado Aurora Colorado USA; ^2^ Division of Pulmonary and Critical Care, Department of Medicine University of Alabama at Birmingham Birmingham Alabama USA; ^3^ Division of Cardiology, Department of Medicine University of Colorado Aurora Colorado USA; ^4^ Division of Pulmonary and Critical Care, Department of Medicine Tulane University School of Medicine New Orleans Louisiana USA; ^5^ Cardiovascular Pulmonary Research Laboratories and Pediatric Critical Care Medicine, Department of Pediatrics University of Colorado Aurora Colorado USA; ^6^ Division of Pulmonary, Allergy and Critical Care, Department of Medicine University of Pittsburgh Medical Center Pittsburgh Pennsylvania USA; ^7^ John W. Deming Department of Medicine Tulane University School of Medicine New Orleans Louisiana USA

**Keywords:** cellular senescence, fibroblast, fibrosis, myofibroblast, oxidative stress, UCP2, uncoupling protein‐2

## Abstract

Mitochondrial dysfunction has been associated with age‐related diseases, including idiopathic pulmonary fibrosis (IPF). We provide evidence that implicates chronic elevation of the mitochondrial anion carrier protein, uncoupling protein‐2 (UCP2), in increased generation of reactive oxygen species, altered redox state and cellular bioenergetics, impaired fatty acid oxidation, and induction of myofibroblast senescence. This pro‐oxidant senescence reprogramming occurs in concert with conventional actions of UCP2 as an uncoupler of oxidative phosphorylation with dissipation of the mitochondrial membrane potential. UCP2 is highly expressed in human IPF lung myofibroblasts and in aged fibroblasts. In an aging murine model of lung fibrosis, the in vivo silencing of UCP2 induces fibrosis regression. These studies indicate a pro‐fibrotic function of UCP2 in chronic lung disease and support its therapeutic targeting in age‐related diseases associated with impaired tissue regeneration and organ fibrosis.

## Graphical abstract

High constitutive levels of uncoupling protein‐2 (UCP2) are observed with aging and in the age‐related fibrotic disease, idiopathic pulmonary fibrosis (IPF). UCP2 functions as an exporter of fatty acid (FA) anions, which may account for the observed decrease in mitochondrial membrane potential of IPF myofibroblasts. Decreased coupling efficiency and fatty acid oxidation (FAO) lower ATP synthetic capacity in association with increased reactive oxygen species (ROS) production. These alterations promote induction of senescence and apoptosis resistance of myofibroblasts, hallmarks of recalcitrant and progressive fibrosis as seen in IPF.

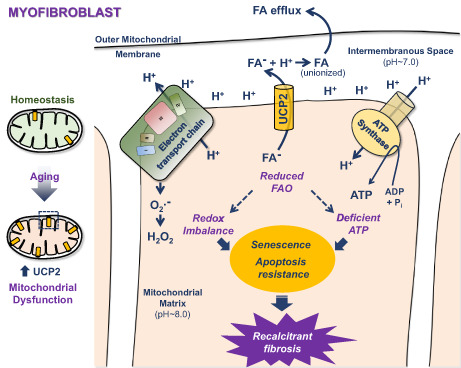

## 1 INTRODUCTION

Fibrosis occurs in multiple pathological conditions and is typically associated with an inadequate or failed regenerative response to tissue injury (Duffield et al., 2013; Horowitz & Thannickal, 2019; Thannickal et al., 2004). Aging is an important contributor of failed tissue regeneration. The cellular and molecular mechanisms that account for this loss of regenerative capacity are not well understood. While a number of so‐called aging “hallmarks” such as stem‐cell exhaustion, cellular senescence, deregulated nutrient‐sensing, and mitochondrial dysfunction have been implicated (Lopez‐Otin et al., 2013; Rangarajan et al., 2017), how these may be integrated to explain key cellular, tissue and organ‐level phenotypes are largely unknown. Idiopathic pulmonary fibrosis (IPF) is a chronic, progressive fibrosing disease of the lungs with increasing incidence and prevalence with age (Raghu et al., 2006). Susceptibility to pulmonary fibrosis with aging has been linked to mitochondrial dysfunction (Mora et al., 2017), oxidative stress (Hecker et al., 2014), and metabolic derangements (Bueno et al., 2020; Romero et al., 2016), but cause–effect relationships remain unclear.

Mitochondrial uncoupling protein‐2 (UCP2) belongs to the SLC25 family of mitochondrial anion carrier proteins (Brand & Esteves, 2005). Although UCP2 has been demonstrated to regulate cellular energy homeostasis along with glucose and fatty acid (FA) metabolism (Pecqueur et al., 2008; Rousset et al., 2004; Vozza et al., 2014), its exact physiological role remains unknown. While many studies implicate a role for UCP2 as a stress‐inducible protein that mediates antioxidant effects (Brand & Esteves, 2005; Mailloux & Harper, 2011), its participation in aging and pathological fibrosis has not been well studied. In this study, we explored the role of UCP2 in cellular bioenergetics, mitochondrial reactive oxygen species (ROS) production, and fatty acid oxidation (FAO) in lung fibroblasts. We uncovered an unanticipated effect of the chronic elevation of this stress‐inducible protein in myofibroblast redox deregulation, differentiation, senescence, and apoptosis resistance that contribute to persistent, non‐resolving lung fibrosis associated with aging.

## 2 RESULTS

### 2.1 UCP2 is highly expressed in IPF lungs and (myo)fibroblasts

**FIGURE 1:**
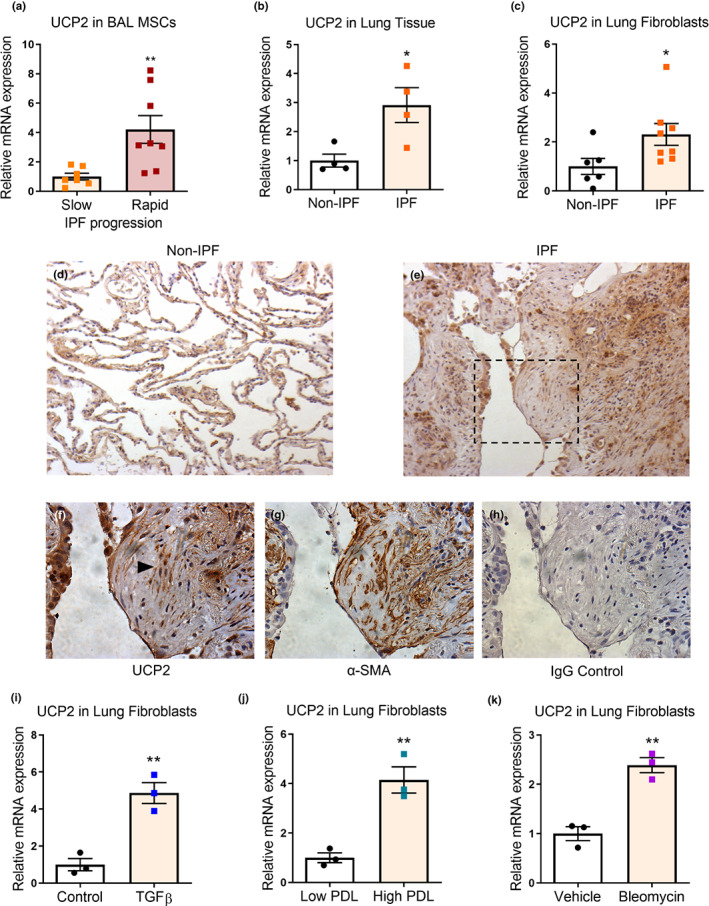
UCP2 is highly expressed in IPF lungs and (myo)fibroblasts. (a) Mesenchymal stromal cells (MSCs) were isolated and cultured from broncho‐alveolar lavage (BAL) fluid of patients with stable (FVC loss ≤5% in the preceding 6 months) or progressive (FVC loss >10% in the preceding 6 months) IPF. qPCR was performed to assess the expression of UCP2. Graph represents mean ± SEM (*n* = 7–8); **p < 0.01. (b) Whole lung tissue obtained from explants of rejected donor lungs (non‐IPF) and IPF lungs were assessed for UCP2 expression by qPCR. Graph represents mean ± SEM (*n* = 4); *p < 0.05. (c) Fibroblasts were isolated from explants of rejected donor lungs (non‐IPF) and IPF lungs and cultured ex vivo. qPCR was performed to assess the expression of UCP2. Graph represents mean ± SEM (*n* = 6–8); *p < 0.05. (d–h) Immunohistochemical staining for UCP2 was performed on non‐IPF and IPF lung sections. Representative images (20× magnification) are shown in (d,e), respectively. A fibroblastic focus was identified in the IPF lung in (e) (dotted rectangle); (f–h) high magnification (40×) view of the fibroblastic focus stained with UCP2, α smooth muscle actin (α‐SMA) and secondary IgG antibody control, respectively. Black arrowhead in (f) shows fibroblasts in the fibroblastic focus. (i) Serum‐starved human diploid lung (IMR‐90) fibroblasts were treated with transforming growth factor‐β1 (TGF‐β1) 2 ng/ml for 24 h. UCP2 expression was assessed by real‐time PCR. Graph represents mean ± SEM (*n* = 3); **p < 0.01. (j) IMR‐90 fibroblasts at low population doubling length (low PDL; PDL < 20), and those undergoing replicative senescence at high PDL (PDL > 40) were assessed for UCP2 expression by real‐time PCR. Graph represents mean ± SEM (*n* = 3); **p < 0.01. (k) Non‐IPF human lung fibroblasts were treated with bleomycin 25 μg/ml for 72 h. UCP2 expression was assessed by real‐time PCR. Graph represents mean ± SEM (*n* = 3); **p < 0.01

In a transcriptomic analysis of mesenchymal stromal cells (MSCs) isolated from bronchoalveolar lavage of human subjects with IPF, we observed increased expression of UCP2 in individuals with progressive vs. stable IPF and confirmed this higher expression of UCP2 in these cells grown in vitro (Figure 1a). For this analysis, progressive disease was defined as a loss in forced vital capacity (FVC) of greater than 10% and stable disease as FVC loss ≤5% in the preceding 6 months, as determined by pulmonary function testing. We validated this finding in whole lung tissue and fibroblasts isolated from explanted lungs of individuals undergoing lung transplantation; IPF subjects demonstrated significantly higher UCP2 gene expression compared to non‐IPF controls (Figure 1b,c). Furthermore, a publicly available dataset of human lung tissues from control subjects and both early and advanced IPF revealed the highest expression of UCP2 in advanced IPF (GSE24206) (Meltzer et al., 2011) (Figure S1a).

To determine the cellular localization of UCP2, we performed immunohistochemistry on tissue sections of IPF subjects and found higher expression in regions of active fibrosis in comparison to normal lungs (Figure 1d,e). In fibroblastic foci that are a hallmark of IPF histopathology, the vast majority of fibroblasts within these foci express UCP2 and the myofibroblast marker, α‐smooth muscle actin (α‐SMA) (Figure 1f–h). Transforming growth factor‐β1 (TGF‐β1) serves as a central mediator of myofibroblast differentiation and fibrogenesis in vivo (Thannickal et al., 2003). Exogenous stimulation of human lung fibroblasts with TGF‐β1 induced a greater than 4‐fold induction of UCP2 mRNA at 24 h following treatment (Figure 1i). Higher expression of UCP2 mRNA was also observed in human lung fibroblasts subjected to undergo replicative senescence with a population doubling length >40 (Figure 1j.). Additionally, treatment of non‐IPF lung fibroblasts with bleomycin for 72 h induced a robust expression of UCP2 (Figure 1k). An increase in UCP2 gene expression was also observed in senescent fibroblasts, either replication‐induced or following bleomycin treatment, when compared to non‐senescent fibroblasts in a publicly available dataset (GSE13330) (Pazolli et al., 2009) (Figure S1b). Interestingly, although UCP2 is ubiquitously expressed in different cells/tissues, the lung is among the highest expressing organs (https://www.gtexportal.org/home/gene/UCP2; Figure S1c). In addition, interrogation of a publicly available single‐cell transcriptomics dataset that characterizes fibroblasts in various disease conditions (fibroXplorer) revealed that UCP2 is preferentially expressed in a subset of fibroblasts with increased frequency in IPF (Figure S1d–h). Together, these data indicate that UCP2 is highly expressed in the human fibrotic lung disease, IPF, in senescent (myo)fibroblasts and is inducible by the pro‐fibrotic mediator, TGF‐β1.

### 2.2 UCP2 uncouples oxidative phosphorylation and decreases ATP synthesis

**FIGURE 2:**
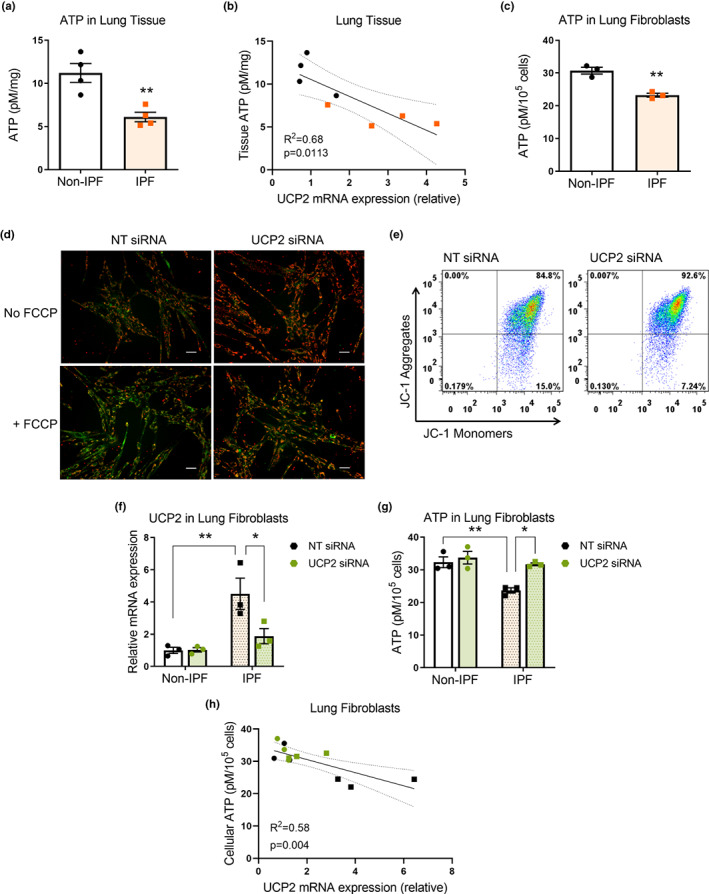
UCP2 uncouples oxidative phosphorylation and decreases ATP synthesis. (a) Whole lung homogenates from non‐IPF and IPF lungs were assessed for ATP content. Graph represents mean ± SEM (*n* = 4); **p < 0.01. (b) Graphical representation of the correlation between ATP content and UCP2 mRNA expression in lung homogenates. (c) Non‐IPF and IPF lung fibroblasts grown ex vivo were assessed for ATP content. Graph represents mean ± SEM (*n* = 3); **p < 0.01. (d) IMR‐90 fibroblasts were treated with non‐targeting (NT) or UCP2‐targeting siRNA for 72 h and incubated with JC‐1 dye (2.5 μg/ml) for 30 min, with/without prior treatment with carbonyl cyanide‐4‐(trifluoromethoxy) phenylhydrazone (FCCP) (5 μM, for 30 min prior to JC‐1) for negative control. Orange/green fluorescence (indicative of JC‐1 aggregates/monomers) was captured by fluorescence microscopy. Scale bars = 50 μm. (e) IPF fibroblasts subjected to siRNA‐mediated knockdown of UCP2 for 72 h were incubated with JC‐1 dye (10 μg/ml) for 10 min, and orange/green fluorescence was analyzed by flow cytometry. The representative graphs depict the percentage of fibroblasts over threshold intensity of orange fluorescence on Y axis, with NT siRNA, 85.44 ± 0.8% vs. UCP2 siRNA, 92.12 ± 0.28%; mean ± SEM; *p* < 0.01; *n* = 5 replicates per group; 20,000 events recorded for each replicate. (f–h) Lung fibroblasts isolated and cultured from explants of 3 non‐IPF and 3 IPF subjects were subjected to siRNA‐mediated knockdown of UCP2 for 72 h. Real‐time PCR was performed to assess UCP2 mRNA expression (f); graph represents mRNA expression relative to non‐IPF cells treated with NT siRNA; mean ± SEM (*n* = 3); **p < 0.01, *p < 0.05. In parallel, ATP content was assessed in these fibroblasts (g); graph represents mean ± SEM (*n* = 3); **p < 0.01, *p < 0.05. (h) Graphical representation of correlation between ATP content and UCP2 mRNA expression in these fibroblasts

Despite its name, there is a lack of clarity and debate regarding how UCP2 functions as an “uncoupler” of mitochondrial electron transport from ATP synthesis. To explore this in IPF and aging, we first determined if high levels of UCP2 correlate with low levels of ATP in isolated cells and in tissues. ATP content in lung tissues was found to be markedly reduced in IPF compared with non‐IPF controls (Figure 2a) and was inversely correlated with UCP2 mRNA levels (Figure 2b). Furthermore, (myo)fibroblasts isolated from IPF lungs demonstrated significantly lower ATP content than non‐IPF lung fibroblasts when measured on a per cell basis (Figure 2c). To determine whether UCP2 contributed to the decreased levels of ATP in lung fibroblasts, we designed an siRNA sequence that was effective in knocking down UCP2 in human lung fibroblasts (Figure S2a–c); orthologous sequences efficiently knocked down UCP2 mRNA in mouse lung fibroblasts and rat lung epithelial cells (Figure S2d,e); greater efficiency of siRNA‐mediated knockdown was observed in IPF fibroblasts (with higher baseline mRNA expression) than in non‐IPF fibroblasts (with lower baseline mRNA expression) (Figure S2a). As a result of an uncoupling effect, UCP2 would be expected to lower the mitochondrial membrane potential (δψm), while UCP2 silencing should, at least partially, reverse this effect. Indeed, IPF lung fibroblasts subjected to UCP2 silencing demonstrated higher δψm, as evidenced by more intense formation of JC‐1 aggregates (Figure 2d,e). Next, we assessed whether UCP2 silencing reverses the energy deficit in IPF lung fibroblasts. In IPF fibroblasts that harbor higher levels of UCP2 and lower ATP content, UCP2 silencing led to marked recovery in ATP synthesis; this effect was not observed in non‐IPF fibroblasts with lower baseline levels of UCP2 (Figure 2f,g). However, in both groups of fibroblasts, cellular ATP content inversely correlated with UCP2 mRNA (Figure 2h). Thus, UCP2 appears to function as an uncoupler of oxidative phosphorylation in IPF lung (myo)fibroblasts, and its silencing increases ATP synthetic capacity.

### 2.3 Constitutive high‐level expression of UCP2 impairs fatty acid oxidation and alters cellular redox state

**FIGURE 3:**
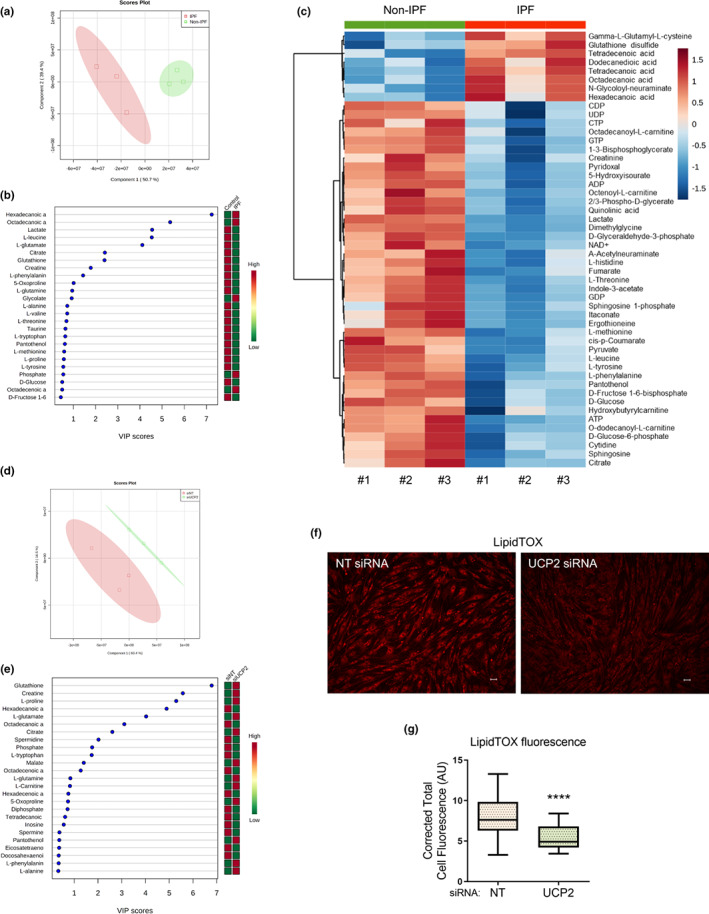
Constitutive high‐level expression of UCP2 impairs fatty acid oxidation and alters cellular redox state. (a–c) Non‐IPF and IPF lung fibroblasts (derived from three explants each) were cultured ex vivo and lysates were subjected to metabolomics analyses. (a) Partial least squares discriminant analysis (PLS‐DA) shows significant separation between the two groups of fibroblasts, (b) variable importance in projection (VIP) scores showing the top 25 metabolites that contribute to the PLS‐DA model, and (c) heatmap of the top 50 metabolites that are significantly different between the two groups; metabolites with lower concentrations are in blue and those with higher concentrations are in red. (d,e) IPF fibroblasts were subjected to siRNA‐mediated knockdown of UCP2 for 24 h. Lysates were subjected to metabolomics analyses. (d) PLS‐DA shows significant separation between the fibroblasts treated with non‐targeting siRNA (siNT) and UCP2‐targeting siRNA (siUCP2); (e) VIP scores showing the top 25 metabolites that contribute to the PLS‐DA model. (f) IPF fibroblasts were subjected to siRNA‐mediated knockdown of UCP2 for 72 h, stained with LipidTOX™ Red Neutral Lipid Stain and immunofluorescence imaging performed (representative images shown). Scale bars = 50 μm. (g) Quantification of LipidTOX™ fluorescence [of the fibroblasts in (f)] was performed by randomly selecting 25 individual fibroblasts each in NT siRNA and UCP2 siRNA groups and assessing their corrected total cellular fluorescence, CTCF [CTCF = Integrated density−(Area of selected cell × Mean fluorescence of background readings)], depicted graphically; boxes represent median and extend from 25th to 75th percentiles, and whiskers represent minimum to maximum values in arbitrary units (AU), *n* = 25, ****p < 0.0001. NT, non‐targeting

Based on our findings that constitutive UCP2 mediates oxidative phosphorylation uncoupling and decreases ATP production, we explored potential alterations in cellular metabolism of IPF when compared to non‐IPF control lung fibroblasts with targeted metabolomics analyses. We confirmed lower levels of ATP in IPF (myo)fibroblasts by this mass spectrometric approach in association with a marked shift in concentrations of free fatty acids (FAs); additionally, alteration in cellular redox state was indicated by low levels of the reduced form of the thiol‐containing tripeptide, glutathione (GSH) (Figure 3a–c). To determine if these metabolic perturbations in IPF lung fibroblasts were related to high basal levels of UCP2, we silenced UCP2 for 24 h by siRNA before subjecting them to the same targeted metabolomics analysis. UCP2‐silenced IPF lung fibroblasts showed reversal of the effects on fatty acid utilization/oxidation (FAO) and redox state, as indicated by lower levels of several free FAs and higher levels of reduced glutathione (Figure 3d,e; Figure S3). The suppressive effects of UCP2 on FAO were confirmed by findings of lower free FAs in the supernatant of UCP2‐silenced cells by mass spectrometry (data not shown), as well as the intracellular accumulation of neutral lipids by LipidTox staining (Figure 3f,g). Taken together, these data indicate that deficient free‐FA utilization/oxidation and heightened oxidative stress in IPF lung fibroblasts are mediated by UCP2.

### 2.4 Chronic elevation of UCP2 induces increased production of reactive oxygen species in IPF lung fibroblasts

**FIGURE 4:**
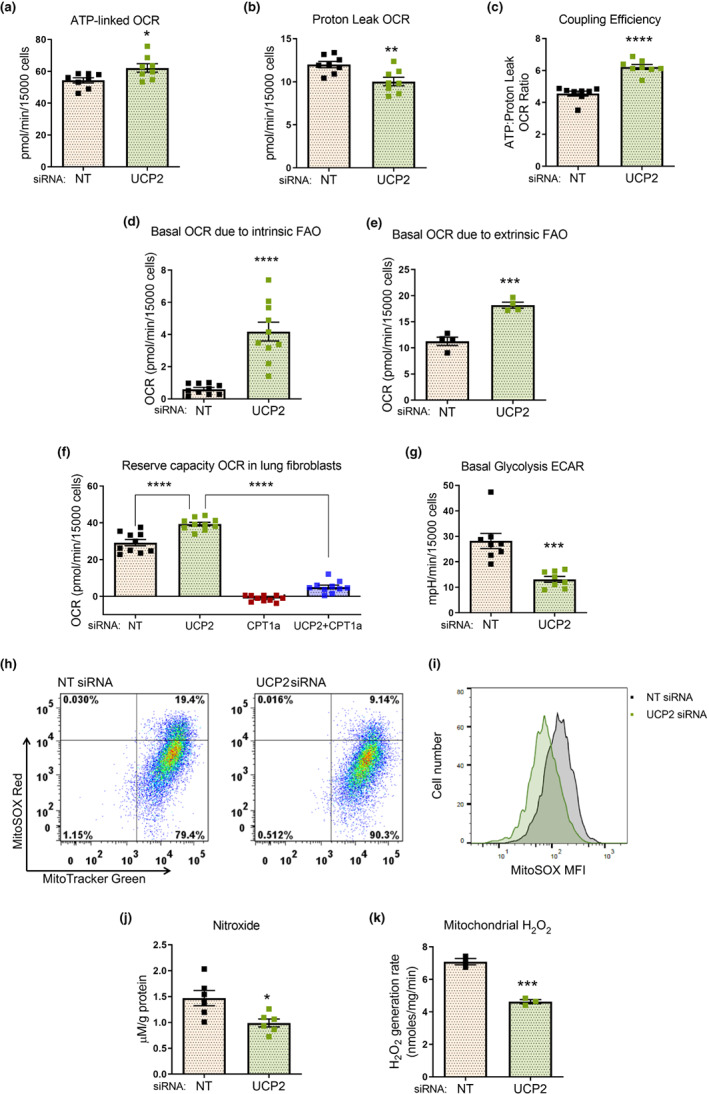
Chronic elevation of UCP2 induces increased production of reactive oxygen species in IPF lung fibroblasts. (a–c) IPF fibroblasts were subjected to siRNA‐mediated silencing of UCP2 for a total of 72 h. The fibroblasts were seeded 15,000 cells per well prior to measurement of oxygen consumption rate (OCR) in an XFe96 analyzer (Seahorse Bioscience); computed results for ATP‐linked OCR (a), Proton Leak OCR (b), and their ratio depicting the coupling efficiency (c) depicted graphically. Error bars represent mean ± SEM (*n* = 8); *p < 0.05, **p < 0.01, ****p < 0.0001. (d) IPF fibroblasts were subjected to siRNA‐mediated silencing of UCP2 for a total of 72 h, seeded at 15,000 cells per well, incubated with a substrate‐restricted medium and treated with etomoxir 4 µM or vehicle. Basal OCR due to intrinsic fatty acid oxidation (FAO) was calculated based on the difference between the OCR just prior to treatment with etomoxir and the OCR 30 min after treatment with etomoxir (depicted graphically). Error bars represent mean ± SEM (*n* = 10); ****p < 0.0001. (e) IPF fibroblasts were subjected to siRNA‐mediated silencing of UCP2 for a total of 72 h, seeded at 15,000 cells per well, incubated with a substrate‐restricted medium and treated with bovine serum albumin (BSA) alone or with BSA‐Palmitate conjugate as per the kit manufacturer's instructions. Basal OCR due to extrinsic FAO was calculated by the difference between the basal OCR of Palmitate‐treated cells and the cells treated with BSA alone, and subsequently subtracting the OCR due to excess proton leak in the Palmitate‐treated cells; thus, calculated basal OCR due to extrinsic FAO is depicted graphically. Error bars represent mean ± SEM (*n* = 4); ***p < 0.001. (f) IPF fibroblasts were subjected to siRNA‐mediated silencing of UCP2, or CPT1a, or both, for a total of 72 h. The fibroblasts were seeded 15,000 cells per well and incubated in a substrate‐restricted medium prior to measurement of OCR; computed results for reserve capacity depicted graphically. Error bars represent mean ± SEM (*n* = 10); ****p < 0.0001. (g) IPF fibroblasts with similar experimental conditions as in (a–c) were assessed for extracellular acidification rate (ECAR) in an XFe96 analyzer; ECAR denoting basal glycolysis depicted graphically. Error bars represent mean ± SEM (*n* = 8); ***p < 0.001. (h) IPF fibroblasts subjected to siRNA‐mediated knockdown of UCP2 for 72 h were incubated with MitoSOX™ Red (5 µM) and MitoTracker™ Green (100 nM) dyes for 15 min, and red–green fluorescence was analyzed by flow cytometry. The representative graphs depict the percentage of fibroblasts over threshold intensity of red fluorescence on Y axis, with NT siRNA, 19.03 ± 1.45% vs. UCP2 siRNA, 8.90 ± 0.38%; mean ± SEM; *p* < 0.0001; *n* = 6 replicates per group; 20,000 events recorded for each replicate. No significant difference noted in green fluorescence between groups. (i) IPF fibroblasts were subjected to siRNA‐mediated knockdown of UCP2 for 72 h. The fibroblasts were incubated with MitoSOX™ Red (5 µM) alone for 15 min, and red fluorescence was analyzed by flow cytometry, with change in intensity depicted graphically and graph representative of 5 replicates. (j) IPF fibroblasts were subjected to siRNA‐mediated knockdown of UCP2 for 24 h. Electron paramagnetic resonance (EPR) spectroscopy was performed with cyclic hydroxylamine spin probes to assess levels of nitroxide formation, reflecting levels of free radicals, chiefly superoxide in the cells (depicted graphically). Graph represents mean ± SEM (*n* = 6); *p < 0.05. (k) IPF fibroblasts were subjected to siRNA‐mediated knockdown of UCP2 for 72 h. Mitochondria were isolated and the rate of hydrogen peroxide production assessed using the p‐hydroxyphenylacetic acid (pHPA) assay; graph represents mean ± SEM (*n* = 3); ***p < 0.001. NT, non‐targeting; CPT1a, carnitine palmitoyl transferase‐1a

To further explore the effects of UCP2 on cellular bioenergetics and oxidative stress in IPF fibroblasts, we measured oxygen consumption rate (OCR) and extracellular acidification rate (ECAR) using an XFe96 extracellular flux analyzer (Seahorse Biosciences, North Billerica, MA). Consistent with our findings on δψm, silencing of UCP2 resulted in higher ATP‐linked OCR and lower proton leak with an increase (either trend or statistically significant) in total (basal) OCR (Figure 4a–c; Figure S4a,b); coincidentally, maximal and reserve capacities were increased under the same conditions. To determine if these cellular bioenergetic shifts were linked to FAO, we first examined whether inhibiting mitochondrial FA uptake with etomoxir (an inhibitor of CPT1a that transports FAs across the mitochondrial membrane, the rate‐limiting step of FAO) influenced the effects of UCP2 silencing. Etomoxir decreased basal OCR of UCP2‐silenced cells to a greater extent than non‐targeting siRNA cells, indicating increased metabolic flux through the FAO pathway in UCP2‐deficient cells (Figure 4d; Figure S4c). A similar pattern of increased OCR linked to FAO was observed with palmitate loading of UCP2‐silenced cells (Figure 4e; Figure S4d). To confirm these findings, we employed a genetic strategy to inhibit FAO by silencing CPT1a. We observed that CPT1a silencing abrogated the effects of elevated reserve capacity seen with UCP2 silencing (Figure 4f; Figure S4e). These studies support inefficient cellular bioenergetics, particularly decreased reserve capacity via FAO, when UCP2 is chronically elevated. In contrast to effects on OCR, basal levels of glycolytic ECAR were reduced with UCP2 silencing (Figure 4g; Figure S4f,g), supporting the concept of lower demand on glycolysis when FAO‐dependent ATP generation is restored.

Based on the observation that IPF fibroblasts generate high levels of oxidized glutathione, an effect reversed by UCP2 silencing, we conducted studies in which the production of ROS was directly measured. We assayed mitochondrial superoxide production using MitoSOX™ staining and flow cytometric analysis; basal levels of superoxide production by mitochondria were found to be reduced by UCP2 silencing without a change in mitochondrial number, mitochondrial DNA content, or the expression of mitochondrial electron transport chain proteins (Figure 4h,i; Figure S4h,i); the decrease in superoxide levels was confirmed with electron paramagnetic resonance studies as measured by production of its reaction product, nitroxide, at early time points after siRNA treatment (Figure 4j). Since superoxide anions are spontaneously or enzymatically reduced to hydrogen peroxide (H_2_O_2_), we also measured H_2_O_2_ production by isolated mitochondria and found a significant reduction in the rate of mitochondrial H_2_O_2_ release with UCP2 knockdown (Figure 4k). Thus, the higher basal expression of UCP2 in IPF fibroblasts is associated with elevated levels of ROS, thereby contributing to oxidative stress and altered cellular redox state.

### 2.5 UCP2 regulates fibroblast senescence and myofibroblast differentiation

**FIGURE 5:**
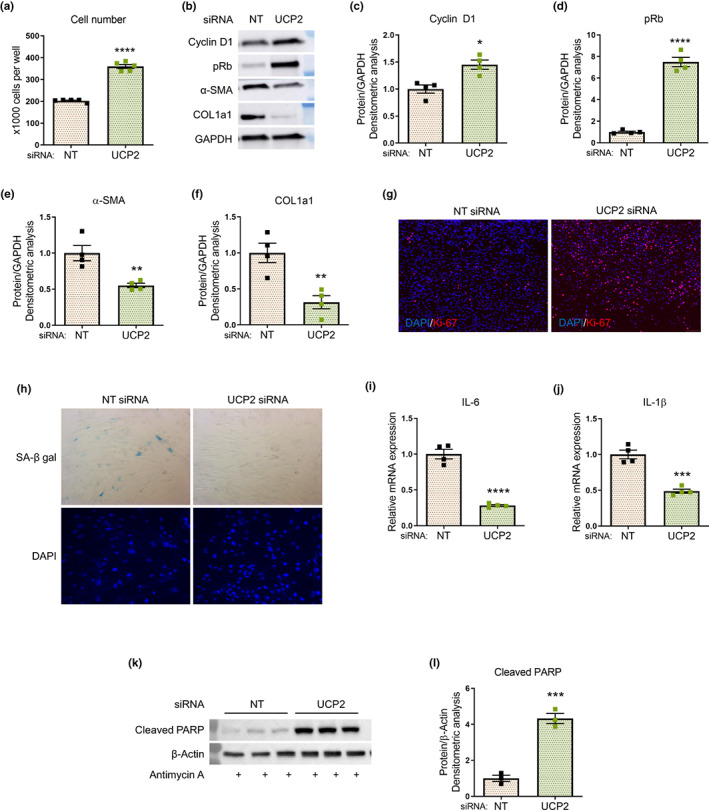
UCP2 regulates fibroblast senescence and myofibroblast differentiation. (a–l) In these experiments, IPF fibroblasts were subjected to siRNA‐mediated silencing of UCP2 for 72 h. (a) Fibroblast cell counting was performed at 72 h (10^5^ cells/well were seeded in both conditions at the start of each experiment); graph represents mean ± SEM (*n* = 5); ****p < 0.0001. (b) Western blotting was performed to assess the steady‐state expression of markers of cell‐cycling, cyclin‐D1 and phosphorylated Rb; myofibroblast markers, α‐smooth muscle actin (α‐SMA) and collagen 1a1 (COL1a1); representative blots are shown; densitometric analyses are shown in (c–f), respectively; graphs represent mean ± SEM (*n* = 4); *p < 0.05, **p < 0.01, ****p < 0.0001. (g) Cells were fixed and stained for DAPI and Ki‐67, a nuclear marker for cell proliferation; immunofluorescence imaging was performed; representative images (10×) are shown. (h) Senescence‐associated β‐galactosidase (SA‐β‐gal) staining with representative light microscopy images (10×) shown. (i,j) Senescence‐associated secretory phenotype (SASP) markers, interleukin 6 (IL‐6) (i) and interleukin 1β (IL‐1β). (j) Gene expression was assessed by real‐time PCR; graphs represent mean ± SEM (*n* = 4); ***p < 0.001, ****p < 0.0001. (k) Antimycin‐A 100 μM was added to the cells for 6 h prior to harvest. Western blotting was performed to assess the steady‐state levels of the apoptosis marker, cleaved poly (ADP‐ribose) polymerase (PARP); densitometric analysis shown in (l); graph represents mean ± SEM (*n* = 3); ***p < 0.001. The effects of UCP2 silencing on these fibroblast phenotypes were confirmed to be similar in fibroblasts derived from lung explants of at least 3 different IPF patients

Oxidative stress has been linked to pulmonary fibrosis (Otoupalova et al., 2020) and to cellular senescence (Hecker et al., 2014). In IPF fibroblasts subjected to UCP2 silencing, we observed an increase in cell proliferation with a concomitant induction of cyclin D1 and phosphorylated Rb, while the expression of myofibroblast differentiation markers, α‐smooth muscle actin (α‐SMA) and collagen 1a1 (COL1a1) was reduced (Figure 5a–f). Enhanced cell proliferation was confirmed using the Ki‐67 staining in the UCP2‐silenced cells (Figure 5g). Furthermore, the expression of senescence‐associated β‐galactosidase (SA‐β‐gal) was reduced in UCP2‐silenced IPF myofibroblasts (Figure 5h), in association with decreased mRNA expression of the senescence‐associated secretory phenotype (SASP) proteins, interleukin 6 (IL‐6) and interleukin 1β (IL‐1β) (Figure 5i,j). The expression of SA‐β‐gal was also reduced in UCP2‐silenced senescent IMR‐90 fibroblasts (Figure S5a). The effects of UCP2 silencing on cellular reprogramming were primarily explained by effects on senescent myofibroblasts which express higher basal levels of α‐SMA and COL1a1 (Figure S5b); a similar effect on the downregulation of these pro‐fibrotic markers was also observed with treatment of IPF myofibroblasts with a pharmacological inhibitor of UCP2, genipin (Zhang et al., 2006) (Figure S5c,d). Senescent myofibroblasts have been well characterized to acquire apoptosis‐resistant properties (Rehan et al., 2021; Zhou & Lagares, 2021). We observed that silencing of UCP2 lowers the apoptosis threshold of these myofibroblasts when stimulated with antimycin A which activates the intrinsic mitochondrial pathway of apoptosis (Figure 5k,l). Together, these data support a critical role for UCP2 in regulating the myofibroblastic, senescent, and apoptosis‐resistant phenotype of IPF fibroblasts.

### 2.6 Therapeutic targeting of UCP2 promotes resolution of experimental lung fibrosis

**FIGURE 6:**
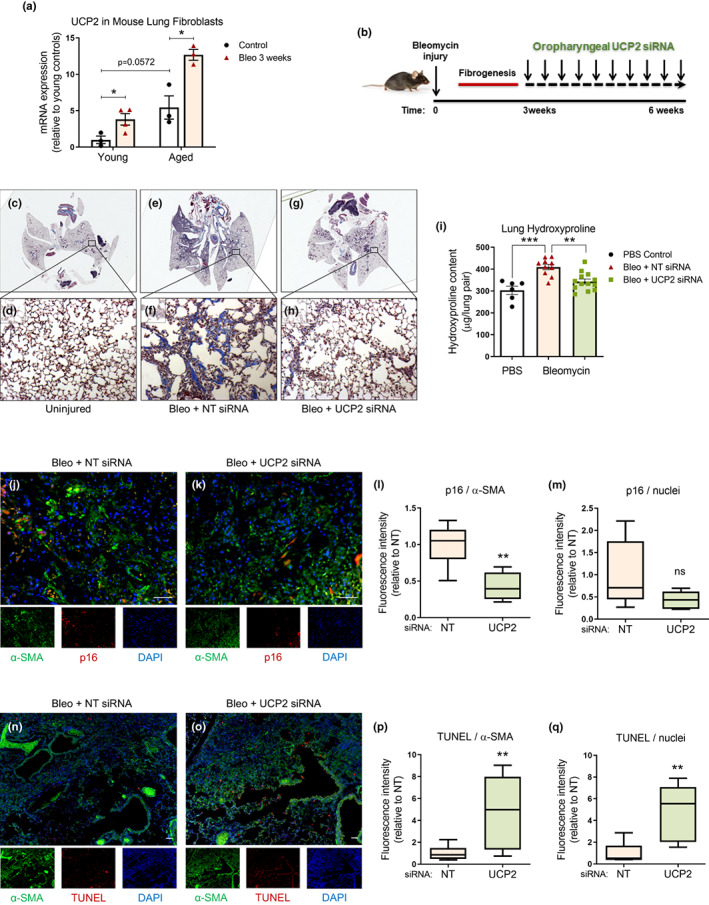
Therapeutic targeting of UCP2 promotes resolution of experimental lung fibrosis. (a) Young (2 months) and aged (18 months old) C57BL/6 mice were subjected to lung injury by instillation of oropharyngeal bleomycin (1.5 U/kg) (or no injury by instillation of PBS control). Lungs were harvested at 3 weeks after bleomycin injury, and fibroblasts were isolated and assessed for gene expression of UCP2 (depicted graphically). Graph represents mean ± SEM (*n* = 3 in each group); *p < 0.05. (b) Schematic depicting experimental design. 18‐month‐old C57BL/6 mice were subjected to lung injury by instillation of oropharyngeal bleomycin (1.5 U/kg) (or no injury by instillation of PBS control). They were treated with UCP2‐targeting or non‐targeting (NT) siRNA, administered oropharyngeally every other day for 3 weeks, starting on day 22 after injury. Lungs were harvested at 6 weeks after injury, and the following analyses were performed. (c–h) Masson's trichrome histochemical staining for collagen was performed. Top panels (c,e,g) show whole lung sections, and bottom panels (d,f,h) show 20× magnification of selected areas. Images are representative of *n* = 3 in each group. (i) Hydroxyproline content of the lungs was assessed (depicted graphically); graphs represent mean ± SEM (*n* = 6–13); **p < 0.01, ***p < 0.001. (j,k) Representative images showing fluorescence patterns of α‐SMA positive fibroblasts (green), senescence marker p16 (red) and nuclei (DAPI‐blue). Scale bars = 50 μm. (l,m) Box plots show fluorescence intensity ratios of p16/α‐SMA and p16/nuclei from regions of enhanced fibrotic remodeling, *n* = 7 per group, 2 mice for each condition. **p < 0.01. (n–q) Representative images show fluorescence patterns of α‐SMA (green), apoptosis marker TUNEL (red) and nuclei (DAPI‐blue). Scale bars = 50 μm. Box plots show relative fluorescence intensity ratios, *n* = 7 per each group, **p < 0.01. NT, non‐targeting; TUNEL, terminal deoxynucleotidyl transferase dUTP nick end labeling

Decreased regenerative capacity and impaired fibrosis resolution are phenotypic characteristics of aged mice (Caporarello et al., 2020; Hecker et al., 2014). Bleomycin‐induced lung injury is commonly used as a rodent model of pulmonary fibrosis, although the influence of aging is seldom accounted for in this model. In this model, oropharyngeal (or intratracheal) instillation of bleomycin typically leads to peak fibrosis at 2–3 weeks post‐injury followed by gradual resolution over several weeks (Izbicki et al., 2002; Moeller et al., 2008). Previous studies, including those by our group, have shown that, despite similar severity of peak fibrosis, resolution of fibrosis is markedly impaired in aged mice (≥18 months) in comparison to young mice (≤2 months) (Hecker et al., 2014; Redente et al., 2011). Using this model, we observed that lung fibroblasts isolated from mice 3 weeks after bleomycin injury showed significant upregulation of UCP2, an effect that was more pronounced in aged mice (Figure 6a). The higher baseline levels of UCP2 in fibroblasts of aged mice were complemented by the publicly available dataset, GSE6591 showing higher UCP2 expression in lungs with increasing age of mice (Misra et al., 2007) (Figure S6a). To test the efficacy of therapeutically targeting UCP2 in established lung fibrosis, we initiated treatment of injured aged mice on day 22 after bleomycin administration (1.5 U/kg) with oropharyngeal UCP2 (or non‐targeting, NT) siRNA (Figure 6b). After 3 weeks of siRNA treatment (and 6 weeks after initial bleomycin injury), we confirmed the efficacy of UCP2 silencing in lung tissue (Figure S6b). At this delayed time point when aged mice fail to resolve fibrosis, we found marked improvement in resolution capacity, as evidenced by histopathology and collagen deposition by Masson's trichrome staining (Figure 6c–h), biochemical measurements of total lung hydroxyproline content (Figure 6i), total lung collagen mRNA (Figure S6c) and static lung compliance (Figure S6d) in mice treated with UCP2 siRNA. The fibrotic areas of the lungs showed evidence of neutrophilic inflammation that was not significantly different between the treatment groups (Figure S6e,f). Importantly, in the mice treated with UCP2 siRNA after bleomycin injury, we observed lower levels of the senescence marker p16 colocalizing with myofibroblasts (expressing high levels of α‐SMA) (Figure 6j–m). The lungs of the mice treated with UCP2 siRNA also demonstrated evidence of higher numbers of apoptotic cells, many of which colocalized with the myofibroblasts (Figure 6n–q). Lung fibroblasts isolated from these mice showed stable reductions in steady‐state levels of the pro‐fibrotic markers, α‐SMA and COL1a1 (Figure S6g–i). In bleomycin‐induced lung injury, there is a general overall correlation between levels of COL1a1 and UCP2 mRNA in isolated lung fibroblasts (Figure S6j). Overall, these results indicate that suppressing expression of UCP2 in aged mice with persistent, non‐resolving fibrosis reprograms the fibrotic phenotype of myofibroblasts to effectively promote fibrosis resolution.

## 3 DISCUSSION

UCPs belong to a subfamily of solute transporters embedded within the inner membrane of mitochondria (Brand & Esteves, 2005). While the physiological function of UCP1 as an uncoupler of oxidative phosphorylation in association with the regulated production of heat in brown adipocytes is well recognized (Argyropoulos & Harper, 2002; Nicholls & Locke, 1984), roles of the ancestral UCP2 and UCP3 homologs remain unclear. UCP2 appears to function as a mild uncoupler and mitigator of mitochondrial ROS production in immune cells (Arsenijevic et al., 2000; Basu Ball et al., 2011). In this report, we demonstrate that the chronic and constitutive elevation of UCP2 in myofibroblasts, paradoxically, mediates pro‐oxidant effects that serve to sustain the differentiation and pro‐senescent phenotype of lung myofibroblasts. Several lines of evidence support this pro‐oxidant effect of UCP2 in lung myofibroblasts. First, mitochondrial superoxide production, as determined by superoxide‐dependent oxidation of the MitoSOX™ fluorophore, was higher in UCP2‐expressing cells. Second, electron paramagnetic resonance studies confirmed the formation of high levels of nitroxide, a superoxide reaction product in these cells. Third, steady‐state release rates of H_2_O_2_, the dismutation product of superoxide anion, were markedly decreased in UCP2‐silenced IPF myofibroblasts. Finally, the relative levels of oxidized vs. reduced glutathione (GSH), a reliable measure of overall cellular redox state, were noted to be higher in IPF myofibroblasts compared to control (when measured by an unbiased mass spectrometry‐based metabolomics approach), and this effect was reversed with silencing of UCP2. Together, these data provide convincing evidence that the high basal levels of UCP2 mediate constitutively higher production of ROS and consequent oxidative stress responses in IPF lung myofibroblasts.

In addition to higher levels of ROS production in the context of heightened UCP2 expression/activity, our studies support a net efflux of FAs and reduced FAO in the mitochondria of senescent myofibroblasts. This is based on our observations of decreased FA consumption in IPF myofibroblasts expressing high levels of UCP2, an effect that was reversed with UCP2 silencing, detected by both mass spectrometry and lipid staining approaches; furthermore, increased basal oxygen consumption in these UCP2‐silenced cells was abrogated with etomoxir, an inhibitor of CPT1a, the rate‐limiting enzyme for FAO. Furthermore, CPT1a silencing reversed the beneficial effects of UCP2 knockdown on mitochondrial reserve capacity. Consistent with recent studies supporting a link between fatty acid flippase activity and proton transport (Berardi & Chou, 2014), we did observe a dissipation of the proton gradient by UCP2. However, we do not have direct evidence that translocation of protons occurs through UCP2. Our data would support a model by which UCP2 functioning as a FA anion transporter dissipates the proton gradient by neutralization of protons in the intermembranous space, thus facilitating net efflux of FAs from the mitochondrial matrix to the cytosol (see Graphical Abstract).

How might the same protein function as “antioxidant” in one context while mediating a “pro‐oxidant” effect in another? The conventional antioxidant effect of UCP2 has been ascribed to its actions of increasing proton leak and dissipating δψm, thereby decreasing reverse electron transport (Arsenijevic et al., 2000). Other studies suggest that UCP2 has no effect on proton leak or ROS production (Kukat et al., 2014). In contrast, our studies clearly indicate an effect of UCP2 on δψm dissipation with *increased* production of ROS. Importantly, we observed an increase in ATP‐linked OCR, coupling efficiency, maximal OCR and reserve capacity despite a marked *decrease* in ROS production when UCP2 is silenced. Thus, under baseline conditions, the high expression and activity of UCP2 in myofibroblasts appear to skew oxygen metabolism towards partially reduced ROS relative to its complete reduction to water per mole of oxygen consumed/reduced. Taken together, this supports the concept that “electron leak” from mitochondrial ETC might occur, not simply due to increased δψm but due to other factors that influence the forward flow of electrons towards more efficient and complete reduction of oxygen at cytochrome *c*. Interestingly, the incorporation of free FAs into the inner mitochondrial membrane may alter membrane fluidity to alter electron transport function that leads to higher ROS production (Schönfeld & Wojtczak, 2007, 2008). While a role for non‐mitochondrial ROS production by UCP2 cannot be completely excluded, we did not observe a statistically significant decrease in non‐mitochondrial OCR with UCP2 knockdown; however, this finding does not exclude participation of non‐ETC dependent sources of ROS, such as those derived from the NADPH oxidase family (Bernard et al., 2014; Thannickal & Fanburg, 1995).

There is substantive evidence linking increased oxidative stress to myofibroblast differentiation and cellular senescence (Cheresh et al., 2013; Hecker et al., 2009; Jain et al., 2013; Velarde et al., 2012; Wiley et al., 2016). The finding that UCP2 silencing was sufficient to reverse these pro‐fibrotic phenotypes supports the concept that constitutive UCP2‐dependent ROS generation reversibly controls these differentiation‐inducing and pro‐senescence programs. An interesting feature of this metabolic reprogramming is the inefficient and/or defective utilization of FAO for energy production in UCP‐expressing IPF myofibroblasts. Interestingly, restoring FAO by genetic or pharmacological methods has been shown to protect mice from tubulointerstitial fibrosis of the kidney (Kang et al., 2015), and UCP2 deficiency has been linked to reduced lipid deposition and ECM accumulation in an ischemia–reperfusion model of kidney fibrosis (Ke et al., 2020). Our studies support the combined regulation of proton leak and FA transport/utilization as an integrated function of UCP2, serving as both an “uncoupler” and a solute/FA transporter.

A notable strength of the therapeutic approach described here is the prospect of targeting the clearance of senescent cells *after* they have been formed, rather than preventing their formation. Indeed, by reducing UCP2 expression in IPF (myo)fibroblasts, we were able to not only reduce senescence but also lower the apoptosis susceptibility of these recalcitrant cells, a strategy that may prove more effective to induce fibrosis regression in established disease (Horowitz & Thannickal, 2019). Except for the spleen and whole blood, UCP2 appears to the most highly expressed in the lung. The lungs are exposed to elevated levels of oxidative stress due to higher ambient oxygen concentration and exposure to environmental toxicants; thus, while adaptive mechanisms may have evolved to protect against such stress, it also renders this organ more susceptible to chronic diseases associated with oxidative stress. However, UCP2 is ubiquitously expressed and, while it may serve protective role in the context of acute and transient stressors (e.g., mitohormesis), chronic upregulation of this stress‐responsive protein may give rise to degenerative tissue responses in multiple organ systems.

## 4 EXPERIMENTAL PROCEDURES

### 4.1 Source of cells

Primary human mesenchymal stromal cells were obtained from bronchoalveolar lavage fluid of patients with IPF at University of Michigan hospital; primary human lung fibroblasts were isolated from failed donor lungs or from healthy parts of lungs curatively resected for cancer (“non‐IPF”) and from explants of patients with IPF undergoing lung transplantation at University of Alabama at Birmingham (UAB), Birmingham, AL, and at University of Colorado Anschutz Medical Campus – all as approved by the respective Institutional Review Boards. The characteristics of subjects from whom the lung fibroblasts were obtained for ex vivo studies are described in the next section. Normal human fetal lung diploid fibroblasts (IMR‐90 cells) were obtained from ATCC.

### 4.2 Subject characteristics

**Table:** 

ID No.	Age (y)	Sex	Condition	Center
3007	55	M	Non‐IPF	UAB
3008	49	M	Non‐IPF	UAB
3013	61	F	Non‐IPF	UAB
15046	65	M	Non‐IPF	UAB
16029	69	M	Non‐IPF	UAB
2026	60	M	IPF	UAB
2032	66	M	IPF	UAB
2041	56	M	IPF	UAB
15044	53	M	IPF	UAB
15061	63	F	IPF	UAB
LTC‐27	69	M	Non‐IPF	U Colorado
LTC‐29	87	M	Non‐IPF	U Colorado
LTC‐50	66	M	Non‐IPF	U Colorado
LTC‐22	45	M	IPF	U Colorado
LTC‐34	64	M	IPF	U Colorado
LTC‐40	68	M	IPF	U Colorado

### 4.3 Cell culture

Fibroblasts were cultured in DMEM, supplemented with 10% fetal bovine serum (FBS), PenStrep (100 units/ml penicillin, 100 μg/ml streptomycin), and were incubated at 37°C in 5% CO_2_ and 95% air. All experiments with primary cells were performed on cells below the eighth passage.

### 4.4 RT PCR

Fibroblasts (or lung tissues) were washed with PBS. The total RNA was extracted using RNeasy mini kit (Qiagen) according to the manufacturer's instructions. Total RNA was reverse transcribed to cDNA using iScript reverse transcription kit (Bio‐Rad). Expression of mRNA of genes of interest was determined by using specific primers, as listed in the Key Resources Table. The real‐time PCRs were performed in a 7300 real‐time PCR system (Applied Biosystems) using a SYBR Green‐based real‐time PCR assay with SYBR Green PCR master mix (Applied Biosystems). Reactions were carried out for 40 cycles. Data are expressed for each target gene normalized to endogenous GAPDH or β‐Actin as 2^−δδCt^, and relative mRNA expression is represented graphically as fold change compared to control condition.

### 4.5 Immunohistochemistry

Paraffin‐embedded lung tissues were cut into 5 μm sections and mounted on glass slides for staining. The sections were subjected to heat‐induced antigen retrieval as described previously (Chanda et al., 2016) followed by processing for immunohistochemical localization of UCP2 or smooth muscle actin in IPF lung sections. Briefly, xylene was used to deparaffinize the tissue sections which were then hydrated through ethanol series and water. Antigen retrieval was performed in a 95°C water bath using citrate buffer (at pH 6.0) followed by quenching of endogenous peroxidases using 3% hydrogen peroxide. The tissue sections were blocked using 5% normal goat serum for 1 h and then incubated in primary antibodies overnight at 4°C. IgG isotype controls (with no primary antibody) were utilized as negative control. The Dako Envision Dual Link System was used for secondary antibody. Colorimetric detection was achieved using the DAB/H_2_O_2_ kit from the Vector Laboratories. Nuclei were counterstained with hematoxylin (Vector Labs). For mouse lung sections, after the antigen retrieval step, Masson's trichrome staining for collagen was performed.

### 4.6 Light and immunofluorescence microscopy

Light and immunofluorescence microscopy was performed using a Keyence BZ‐X700 microscope. The images obtained were processed using Adobe Photoshop CS6.

### 4.7 ATP assay

Pre‐weighed lung tissue (~30 mg) or 0.15 × 10^6^ lung fibroblasts were processed for determination of ATP content using a commercially available kit (Abcam; ab833355) as per the manufacturer's instructions. Briefly, frozen lung tissue or fibroblast cell pellets were resuspended in ice‐cold ATP assay buffer and lysed using Dounce homogenizer. The resulting lysates were clarified via centrifugation at 13,000 ***g*** at 4°C. The supernatant fractions were collected and subjected to the deproteinization procedure via TCA precipitation (Abcam; ab204708). The deproteinized samples were then incubated with required reaction components for 30 min in the dark at room temperature. Fluorescence signals were measured on a microplate reader at Ex/Em = 535/587 nm. To generate a standard calibration curve, serial dilutions of ATP were used. ATP concentrations were calculated from the standard curve data and normalized to the corresponding tissue weight or number of cells.

### 4.8 RNA interference

siRNA (UCP2 targeting and non‐targeting) with sense sequences as described in the Key Resources Table was obtained from Dharmacon (Supporting Information). Fibroblasts were transfected with 100 nM siRNA using Lipofectamine 2000 in OptiMEM medium according to the manufacturer's protocol overnight. This was followed by recovery using DMEM with 10% FBS the next day for all experiments lasting more than 24 h.

### 4.9 Flow cytometry

Fibroblasts were incubated at 37°C with the indicated fluorescent dye(s), as described in the figure legends. The cells were then washed twice with PBS. Data were collected with an LSR‐II flow cytometer (Becton Dickinson) and analyzed with FlowJo software (version 10.6.1; TreeStar).

### 4.10 Metabolomics analysis

Metabolites from frozen cell pellets were extracted at 2 × 10^6^ cells/ml in ice‐cold 5:3:2 MeOH:acetonitrile:water (v/v/v). Extractions were carried out using vigorous vortexing for 30 min at 4°C. Supernatants were clarified by centrifugation (10 min, 18,000 ***g***, 4°C) and 10 μl analyzed using a Thermo Vanquish UHPLC coupled to a Thermo Q Exactive mass spectrometer. Global metabolomics analyses were performed using a 5 min C18 gradient in positive and negative ion modes (separate runs) with electrospray ionization as described (Gehrke et al., 2019; Nemkov et al., 2019). For all analyses, the MS scanned in MS1 mode across the m/z range of 65–950. Peaks were annotated in conjunction with the KEGG database, integrated, and quality control performed using Maven (Princeton University), as described (Nemkov et al., 2015). Analysis of the output was performed using Metaboanalyst version 5.

### 4.11 Mitochondrial stress test

The assay was performed according to Agilent Seahorse Extracellular Analyzer manufacturer's instructions. Briefly, fibroblasts were seeded on a Seahorse XFe96 assay plate at a density of 1.5 × 10^5^ cells per well 24 h prior to the assay. The sensor cartridge was hydrated with XF calibrant overnight, and the fibroblasts were washed with Agilent Base media supplemented with 10 mM glucose, 1 mM sodium pyruvate, and 2 mM L‐glutamine just prior to the assay. The following drugs were injected sequentially: oligomycin 2.5 μg/ml, FCCP 5 μM, rotenone 2 μM + antimycin A 4 μM and 2‐deoxy glucose 50 mM. Oxygen consumption rate and extracellular acidification rate were measured simultaneously.

### 4.12 Electron paramagnetic resonance (EPR) study

Mitochondrial superoxide production was measured by EPR using the mitochondrial spin probe 1‐hydroxy‐4‐[2‐(triphenylphosphonio)‐acetamido]‐2,2,6,6‐tetramethylpiperidine, 1‐hydroxy‐2,2,6,6‐tetramethyl‐4‐[2‐(triphenylphosphonio)acetamido]piperidinium dichloride (mito‐TEMPO‐H). Fibroblasts were subjected to siRNA‐mediated knockdown of UCP2 24 h prior to the EPR measurements. Mito‐TEMPO‐H probe was prepared in deoxygenated 50 mM phosphate buffer. The cells were washed and treated with mito‐TEMPO‐H 0.25 mM in Krebs‐HEPES buffer (KHB) containing 100 μM of a metal chelator DTPA to avoid direct oxidation with metal ion or hydroxyl radical generation by Fenton reaction. The cells were incubated for 50 min at 37°C and then placed on ice and gently scraped. 50 μl of cell suspension was loaded in an EPR capillary tube, and EPR measurements were performed at room temperature using a Bruker EMXnano X‐band spectrometer. EPR acquisition parameters were microwave frequency = 9.6 GHz; center field = 3432 G; modulation amplitude = 2.0 G; sweep width = 80 G; microwave power = 19.9 mW; total number of scans = 5; sweep time = 12.11 s; and time constant = 20.48 ms. The mito‐TEMPO·(nitroxide) radical concentration was obtained by simulating the spectra using the SpinFit module incorporated in the Xenon software of the bench‐top EMXnano EPR spectrometer followed by the SpinCount module (Bruker), as described previously (Elajaili et al., 2019). Total protein content from the analyzed samples were quantified via a Micro BCA Protein Assay kit (Pierce), and nitroxide concentrations were normalized to total protein.

### 4.13 Mitochondrial H_2_O_2_ production rate

Mitochondrial H_2_O_2_ production was determined fluorometrically as described previously (Murthy et al., 2010). Briefly, mitochondria were isolated by lysing the cells in a mitochondria buffer containing 10 mM Tris, pH 7.8, 0.2 mm EDTA, 320 mM sucrose, and protease inhibitors. Lysates were homogenized using a Kontes Pellet Pestle Motor and centrifuged at 2000 ***g*** for 8 min at 4°C. The supernatant was removed and kept at 4°C, and the pellet was lysed, homogenized, and centrifuged again. The two supernatants were pooled and centrifuged at 12,000 ***g*** for 15 min at 4°C. The pellet was then resuspended in mitochondria buffer without sucrose. The isolated mitochondria were incubated in phenol‐red free Hanks' Balanced Salt solution supplemented with 6.5 mM glucose, 1 mM HEPES, 6 mM sodium bicarbonate, 1.6 mM p‐hydroxylphenyl acetic acid (pHPA), and 0.95 μg/ml HRP. Fluorescence of pHPA‐dimer was measured using a spectrofluorometer at Ex/Em = 320/400 nm at 10 min intervals up to 240 min to calculate rate of production of H_2_O_2_.

### 4.14 Western immunoblotting

Cells were lysed in the radioimmune precipitation assay (RIPA) buffer (Sigma‐Aldrich) followed by addition of protease and phosphatase inhibitors. The total protein concentration of the lysates was quantitated using a Micro BCA Protein Assay kit (Pierce). Further RIPA was added to equalize the concentrations of the lysates, and 10× reducing agent along with 4× loading buffer was added to the lysates, followed by incubation at 95°C for 5 min. The samples were then subjected to SDS‐PAGE, and Western immunoblotting was performed as described previously (Desai et al., 2014). Immunoblots were imaged using Amersham Biosciences 600 Imager (GE Healthcare) at UAB and Biorad ChemiDoc XRS+ imager at University of Colorado. Quantification (densitometry) was performed using Fiji software.

### 4.15 Source of mice

Only male mice (C57BL/6) were used for all experiments. Young mice (8–10 weeks age) were purchased from The Jackson Laboratory, Bar Harbor, ME. Aged mice (~17 months) were procured from the National Institute on Aging by Dr. Thannickal. The mice were housed in vivariums at UAB and CU Anschutz on a 12‐h light–dark cycle with access to food and water ad libitum. All experiments were conducted after approval by the UAB and CU Anschutz IACUC.

### 4.16 Murine model of bleomycin‐induced lung fibrosis

Mice were anesthetized with isoflurane followed by oropharyngeal instillation/aspiration of bleomycin (1.5 U/kg) in 60 μl PBS. Lungs were harvested at 3 weeks after bleomycin instillation for assessing fibrotic burden and UCP2 gene expression in tissue/isolated fibroblasts. In experiments involving therapeutic siRNA administration, mice received UCP2‐targeting or non‐targeting (NT) siRNA (50 μg/dose in 60 μl PBS) administered oropharyngeally under isoflurane anesthesia every other day for 3 weeks, starting on day 22 after injury. Lungs were harvested at 6 weeks after injury for assessing end points including hydroxyproline, collagen and UCP2 expression, and for isolating fibroblasts.

### 4.17 Hydroxyproline assay

Mouse lung tissues were dried in 2 ml microcentrifuge tubes at 70°C in a block heater for 48 h and then hydrolyzed in 6 N HCl at 95°C for further 48 h. The tubes were centrifuged at 13,000 ***g*** for 10 min, and the debris‐free supernatant was transferred to fresh tubes for storage prior to hydroxyproline estimation. Fluorometric hydroxyproline assay was performed using a commercially available kit (QuickZyme Biosciences) with hydroxyproline as a standard, as per the manufacturer's instructions.

### 4.18 Quantification and statistical analysis

Graphing and statistical analysis were performed with Graphpad Prism ver 9.1. Unpaired *t* test was used for comparing 2 variables, and anova for three or more variables with multiple comparisons. All data are expressed as mean ± SEM, unless otherwise indicated. *p* < 0.05 were considered statistically significant.

## AUTHOR CONTRIBUTIONS

Conceptualization: SR and VJT; Conduct of experiments and data generation: SR, MLL, DC, AK, DK, JLL, PL, RAB, HE, ESN and JSD; Analysis of data and interpretation: SR, JWZ and VJT; Manuscript preparation and editing: SR and VJT; Resources: SR, OE and VJT.

## CONFLICT OF INTEREST

VJT has consulted in the broad area of pulmonary fibrosis for the following companies: Mistrial Therapeutics, Inc., Boehringer Ingelheim Pharmaceuticals, Inc., United Therapeutics, Blade Therapeutics, Versant Venture, Translate Bio and Sunshine Bio. SR and VJT have initiated the process of filing a patent on therapeutic targeting of UCP2 in fibrotic diseases.

## Supporting information


Figures S1–S6

## Data Availability

The data that support the findings of this study are available from the corresponding author upon reasonable request.
