# Supplementary material for: Mitochondrial uncoupling protein‐2 reprograms metabolism to induce oxidative stress and myofibroblast senescence in age‐associated lung fibrosis
Source: Aging Cell. 2022 Aug 7;21(9):e13674. doi: 10.1111/acel.13674 (PMC9470902; doi:10.1111/acel.13674)
Supplement: Supplementary file 1 — Figures S1–S6 [file ACEL-21-e13674-s001.pdf]

Figure S1

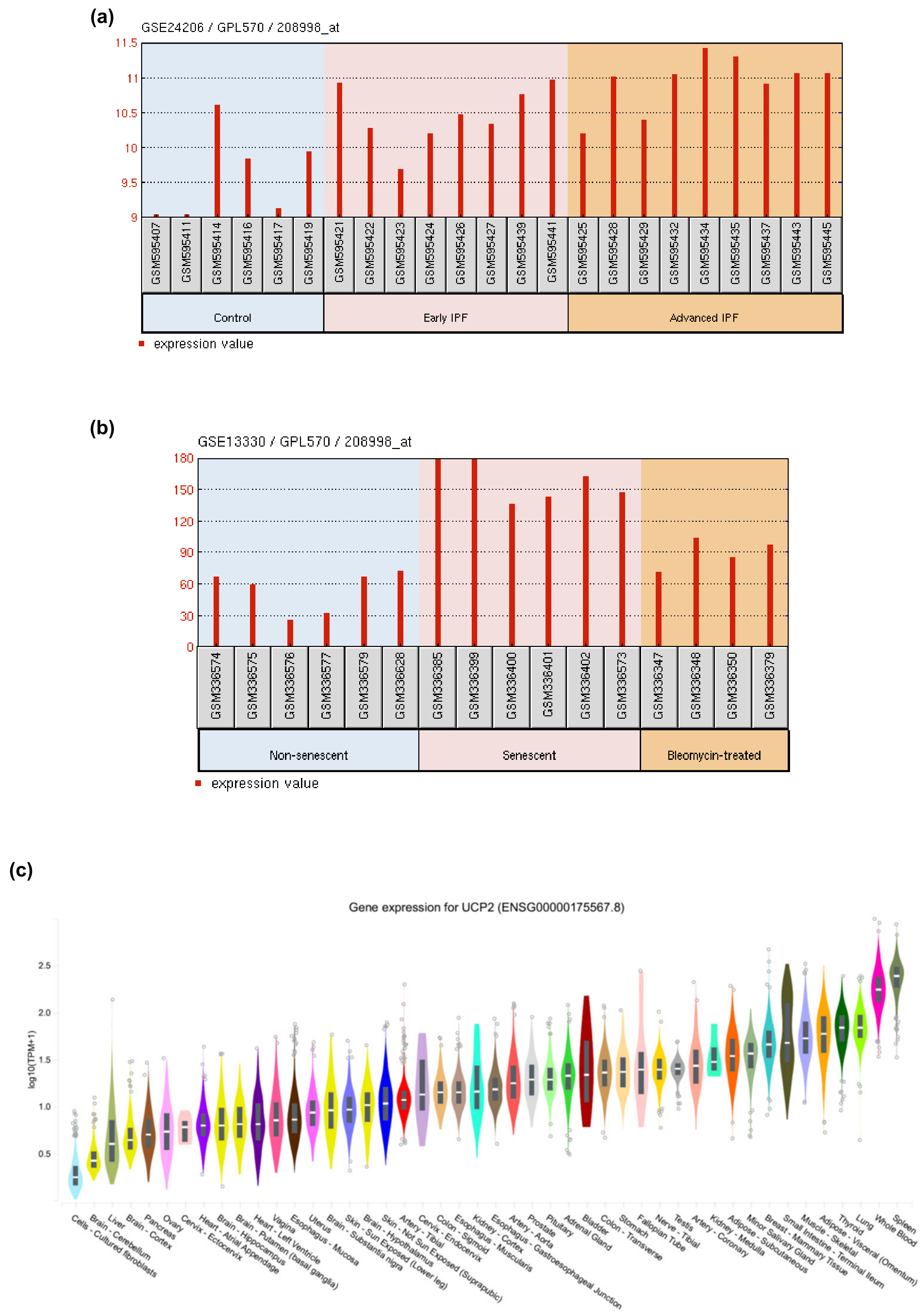
(a)
GSE24206 / GPL570 / 208998_at
11.5
11
10.5
10
9.5
9
GSM595407
GSM595411
GSM595414
GSM595416
GSM595417
GSM595419
GSM595421
GSM595422
GSM595423
GSM595424
GSM595426
GSM595427
GSM595439
GSM595441
GSM595425
GSM595428
GSM595429
GSM595432
GSM595434
GSM595435
GSM595437
GSM595443
GSM595445
Control
Early IPF
Advanced IPF
expression value
(b)
GSE13330 / GPL570 / 208998_at
180
150
120
90
60
30
0
GSM336574
GSM336575
GSM336576
GSM336577
GSM336579
GSM336628
GSM336385
GSM336399
GSM336400
GSM336401
GSM336402
GSM336573
GSM336347
GSM336348
GSM336350
GSM336379
Non-senescent
Senescent
Bleomycin-treated
expression value
(c)
Gene expression for UCP2 (ENSG00000175567.8)
log10(TPM+1)
2.5
2.0
1.5
1.0
0.5
Cells - Cultured fibroblasts
Brain - Cerebellum
Liver
Brain - Cortex
Pancreas
Ovary
Cervix - Ectocervix
Heart - Atrial Appendage
Brain - Hippocampus
Brain - Putamen (basal ganglia)
Heart - Left Ventricle
Vagina
Esophagus - Mucosa
Uterus
Brain - Substantia nigra
Skin - Sun Exposed (Lower leg)
Brain - Hypothalamus
Skin - Not Sun Exposed (Suprapubic)
Artery - Tibial
Cervix - Endocervix
Colon - Sigmoid
Esophagus - Muscularis
Kidney - Cortex
Esophagus - Gastroesophageal Junction
Artery - Aorta
Prostate
Pituitary
Adrenal Gland
Bladder
Colon - Transverse
Stomach
Fallopian Tube
Nerve - Tibial
Testis
Artery - Coronary
Kidney - Medulla
Adipose - Subcutaneous
Minor Salivary Gland
Breast - Mammary Tissue
Small Intestine - Terminal Ileum
Muscle - Skeletal
Adipose - Visceral (Omentum)
Thyroid
Lung
Whole Blood
Spleen

Figure S1 continued…

(d)

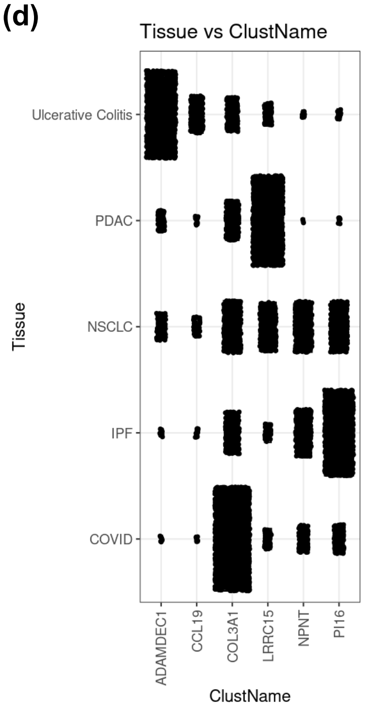

(e)

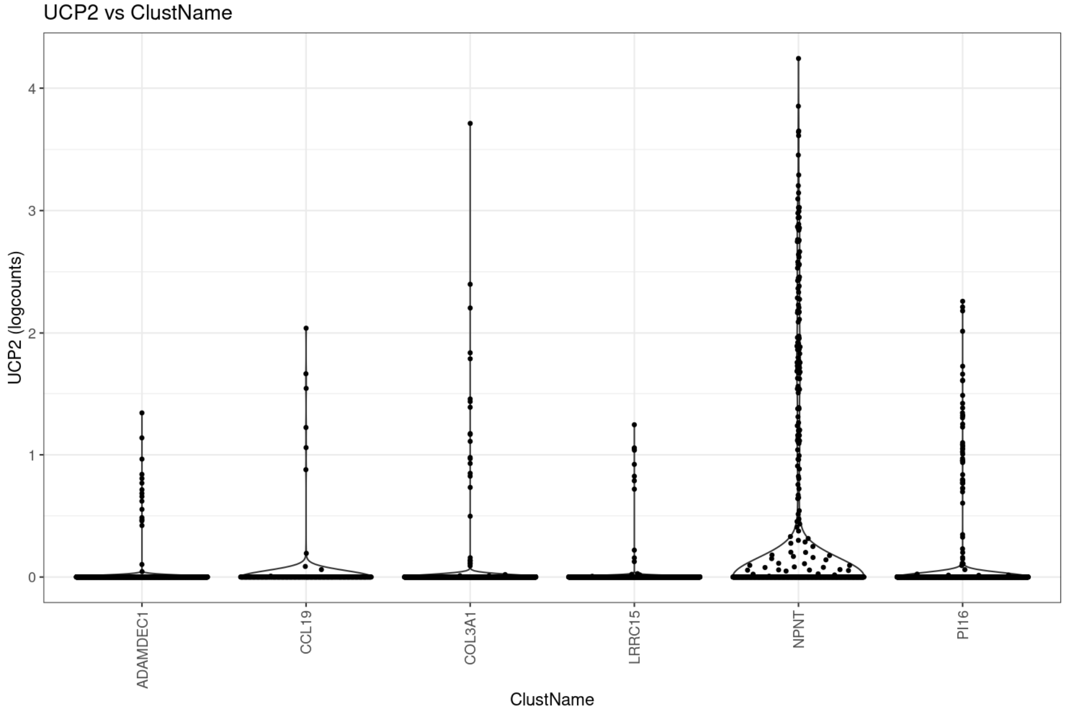

(f)

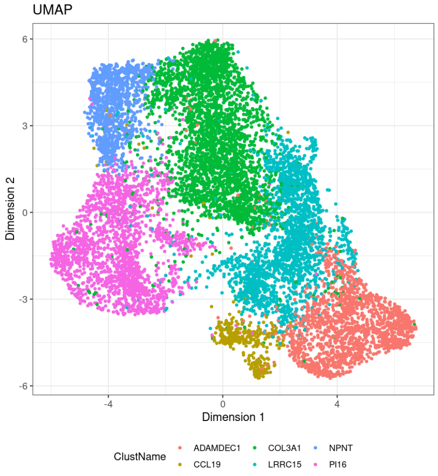

(g)

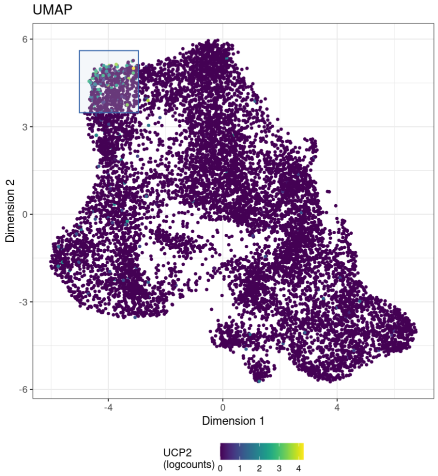

(h)

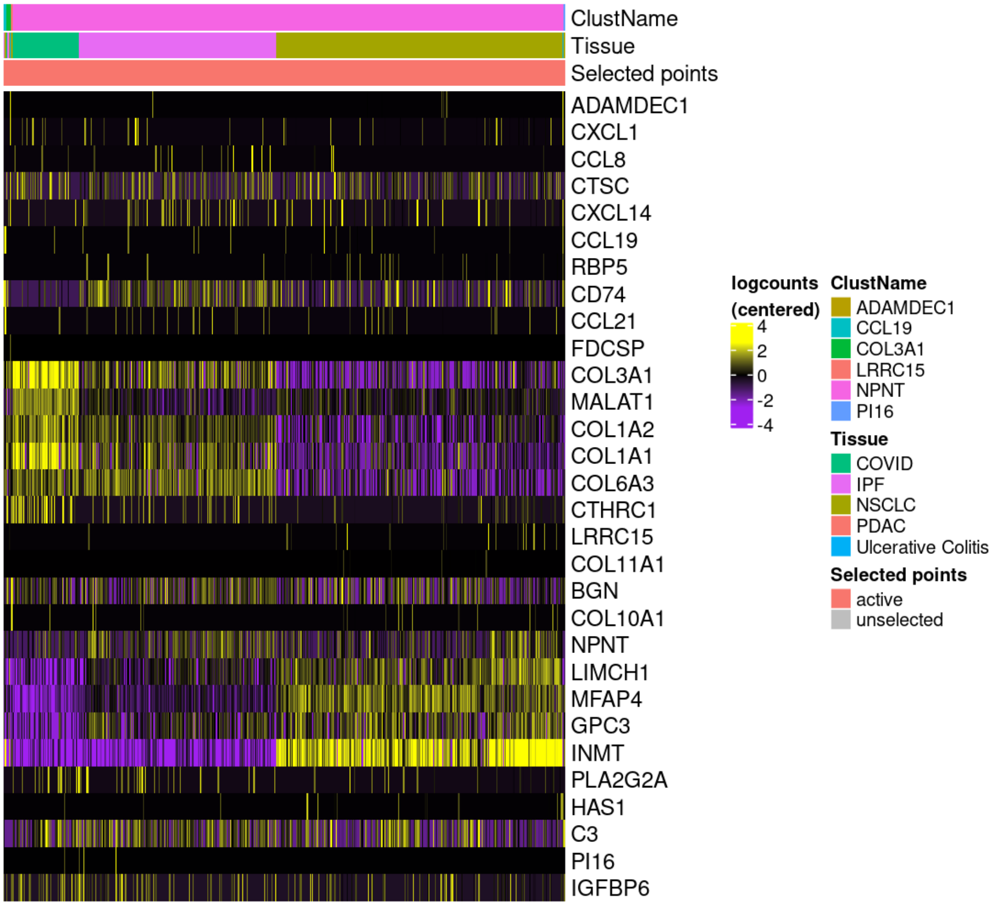

Figure S1. UCP2 is highly expressed in IPF lungs and senescent fibroblasts.

(**a**) A publicly available GEO dataset, GSE24206 (https://www.ncbi.nlm.nih.gov/geo/query/acc.cgi?acc=GSE24206) was queried for UCP2 gene expression in whole lung tissue derived from non-IPF patients and those with early and advanced IPF using the GEO2R tool, graphical output shown. (**b**) A publicly available GEO dataset, GSE13330 (https://www.ncbi.nlm.nih.gov/geo/query/acc.cgi?acc=GSE13330) was queried for UCP2 gene expression in human foreskin fibroblasts that were either non-senescent or subjected to replicative senescence or stress-induced senescence (via exposure to bleomycin) using the GEO2R tool, graphical output shown. (**c**) UCP2 gene expression in various normal tissues in humans was obtained from the Genotype-Tissue Expression (GTEx) project online portal (https://www.gtexportal.org/home/gene/UCP2). The graphical output is in log scale sorted in ascending order of median expression in tissues. (**d-h**) The Fibroexplorer dataset (an online resource tool derived from fibroblast atlases by integration of single-cell transcriptomic data from approximately 230,000 fibroblasts across 17 tissues, 50 datasets, 11 disease states and 2 species) for perturbed state (human), available at https://www.fibroxplorer.com/perturbed-state-human, was queried for UCP2 gene expression; output shown. (**d**) shows the clusters of fibroblasts characterized in the dataset (X axis) in various conditions (Y axis) (ulcerative colitis; PDAC = pancreatic ductal adenocarcinoma; NSCLC = non-small cell lung cancer; IPF = idiopathic pulmonary fibrosis; COVID = covid-19 pneumonia; the latter three fibroblast sets isolated from lungs). The sizes of the black boxes show relative expression of UCP2 in the cluster/condition. (**e**) shows relative expression of UCP2 in the various clusters in violin plots. (**f**) shows the Uniform Manifold Approximation and Projection (UMAP) plot of the various clusters in the dataset, with each cluster showing a different color. (**g**) shows the relative expression of UCP2 among the various clusters in the UMAP plot, with predominant expression in the NPNT cluster. (**h**) shows the relative expression of top genes in the clusters of cells limited by the selection (blue box) in (**g**).

Figure S2

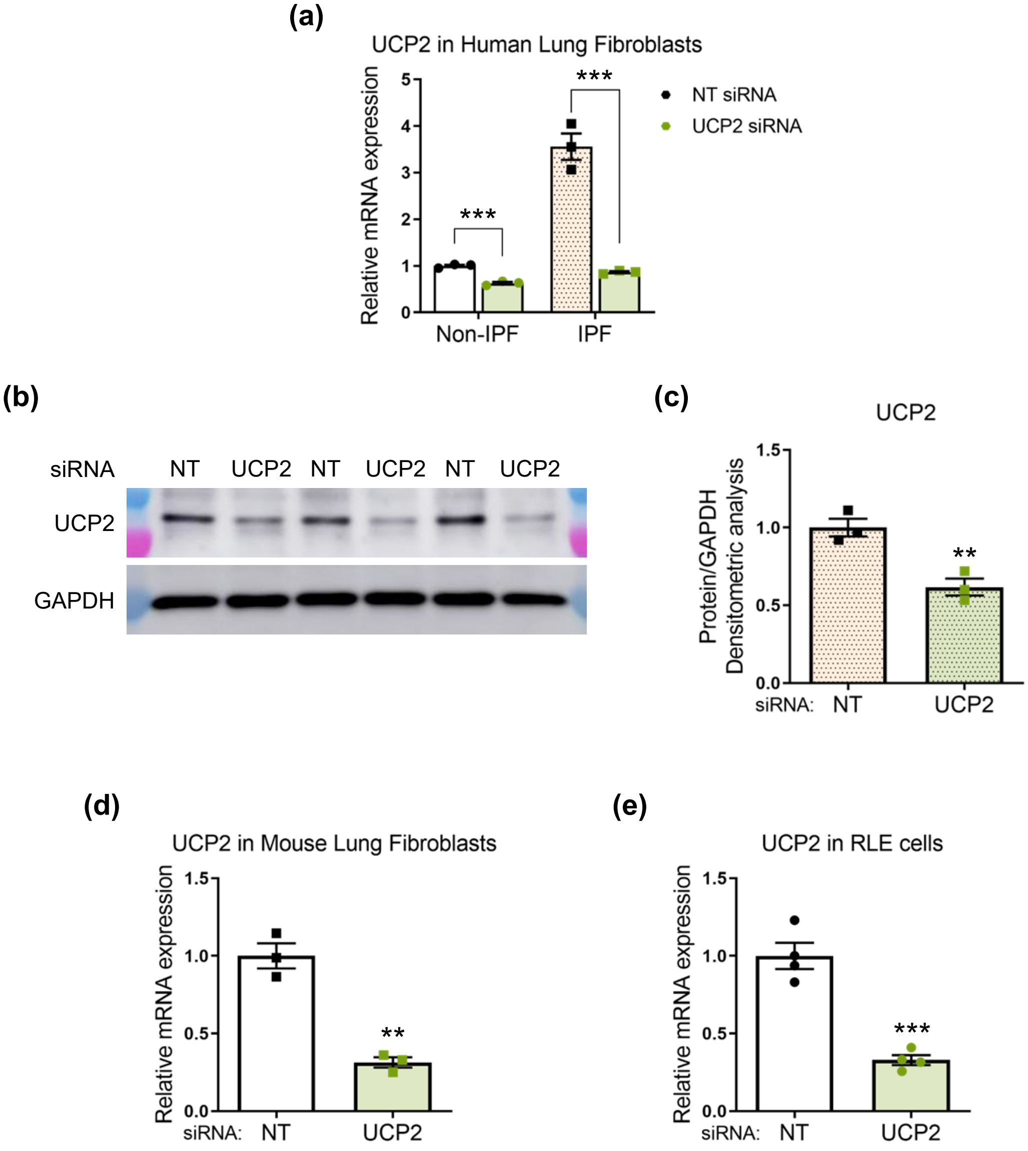

Figure S2. Efficacy of UCP2 siRNA.

An siRNA sequence targeting human UCP2 was designed, and its efficacy tested on non-IPF and IPF fibroblasts (derived from one lung explant each) (**a**). The fibroblasts were treated with the UCP2 siRNA or non-targeting (NT) siRNA (100 nM each) for 72 hours; steady-state mRNA levels assessed by qPCR. Graph represents mean ± SEM (n=3 each group); ***p<0.001. (**b**) IPF fibroblasts were treated with UCP2 or NT siRNA for 72 hours; western blotting was performed to assess steady-state expression of UCP2; representative blots shown, with their densitometric analysis in (**c**); error bars represent mean ± SEM (n=3); **p<0.01. Please note that the UCP2 band appears at ~95 kDa which represents 3x molecular weight of the UCP2 monomer. (**d, e**) A mouse/rat siRNA sequence that is orthologous to the human UCP2 siRNA sequence was generated. This sequence works for both mouse (*Mus musculus*) and rat (*Rattus norvegicus*) and differs from the human sequence in only 2 (out of 19) bases (see Key Resources Table). Mouse lung fibroblasts (**d**) and rat lung epithelial (RLE) cells (**e**) were treated with this siRNA (or non-targeting siRNA) for 72 hours; steady-state mRNA levels assessed by qPCR. Graphs represent mean ± SEM (n=3 each group); **p<0.01, ***p<0.001. NT = non-targeting.

Figure S3

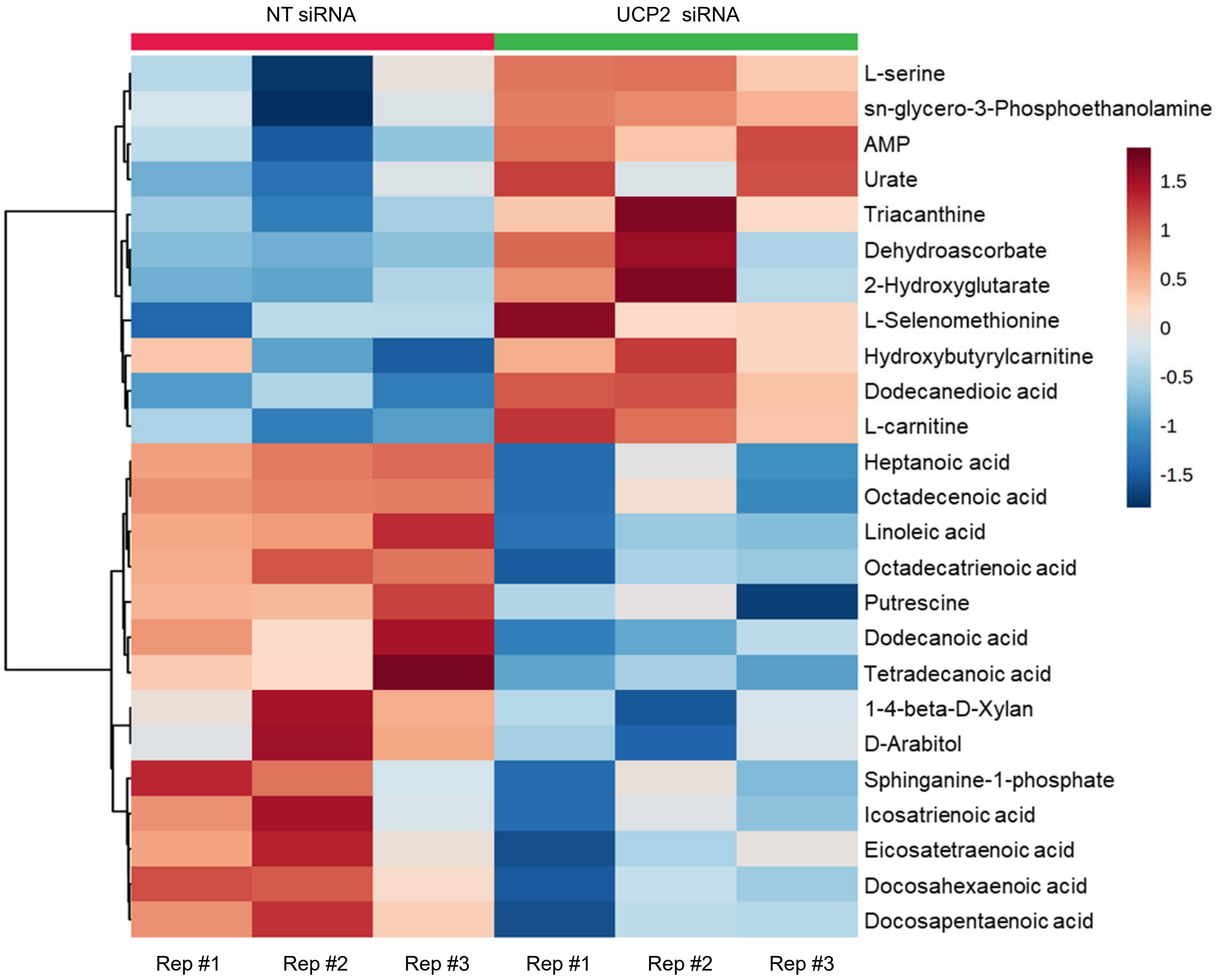

Figure S3. Heatmap of metabolites in IPF fibroblasts treated with UCP2 siRNA.

IPF fibroblasts were subjected to siRNA-mediated knockdown of UCP2 for 72 hours. Lysates (experimental triplicate) were subjected to metabolomics analyses (same experiment described in **Figure 3d, e**). A heatmap of the top 25 metabolites that are significantly different between the two groups is shown; metabolites with lower concentrations are in blue and those with higher concentrations are in red. NT = non-targeting.

Figure S4

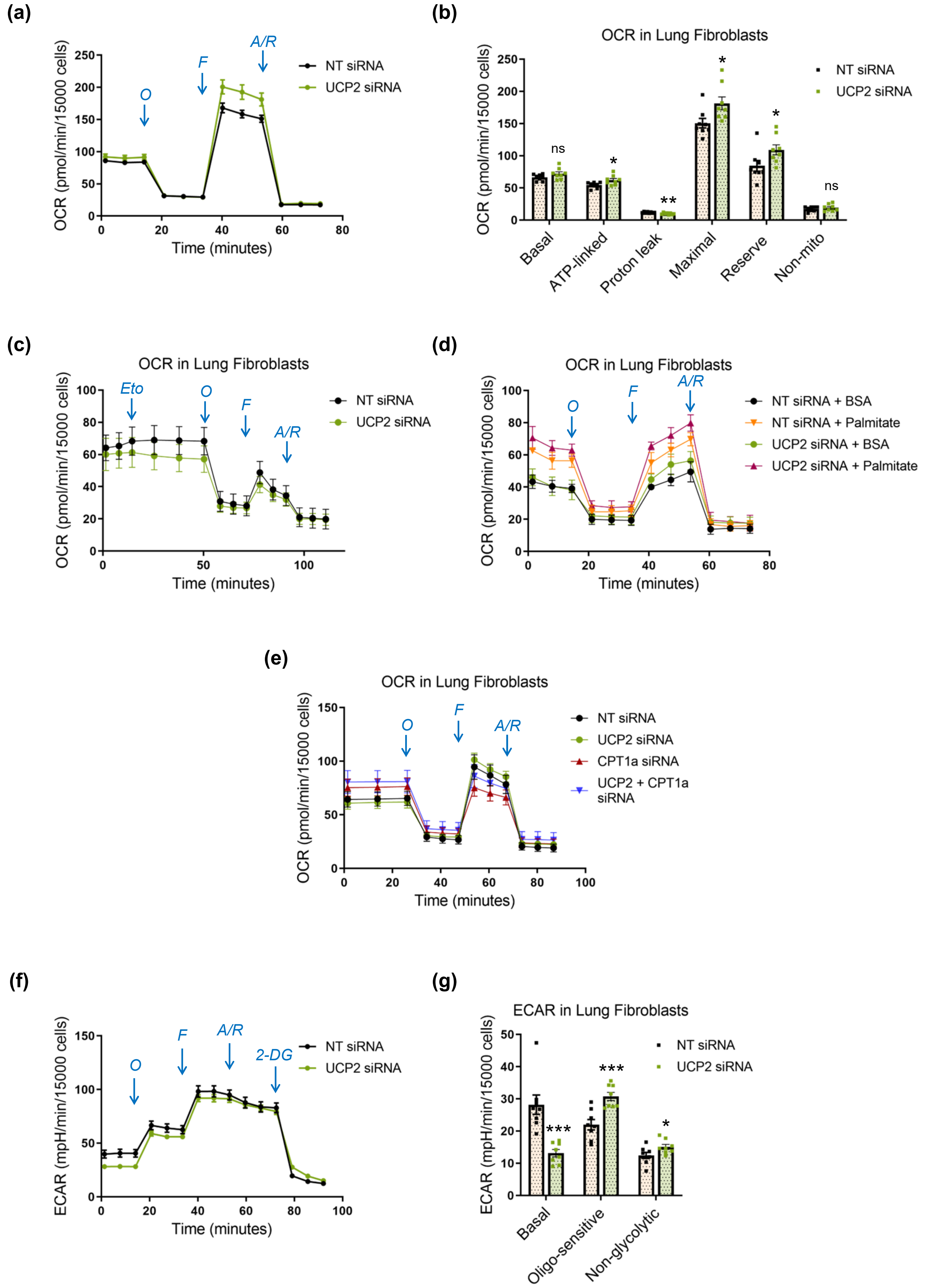
(a)
OCR (pmol/min/15000 cells)
Time (minutes)
O
F
A/R
NT siRNA
UCP2 siRNA
(b)
OCR in Lung Fibroblasts
OCR (pmol/min/15000 cells)
NT siRNA
UCP2 siRNA
ns
*
**
*
*
ns
Basal
ATP-linked
Proton leak
Maximal
Reserve
Non-mito
(c)
OCR in Lung Fibroblasts
OCR (pmol/min/15000 cells)
Time (minutes)
Eto
O
F
A/R
NT siRNA
UCP2 siRNA
(d)
OCR in Lung Fibroblasts
OCR (pmol/min/15000 cells)
Time (minutes)
O
F
A/R
NT siRNA + BSA
NT siRNA + Palmitate
UCP2 siRNA + BSA
UCP2 siRNA + Palmitate
(e)
OCR in Lung Fibroblasts
OCR (pmol/min/15000 cells)
Time (minutes)
O
F
A/R
NT siRNA
UCP2 siRNA
CPT1a siRNA
UCP2 + CPT1a siRNA
(f)
ECAR (mpH/min/15000 cells)
Time (minutes)
O
F
A/R
2-DG
NT siRNA
UCP2 siRNA
(g)
ECAR in Lung Fibroblasts
ECAR (mpH/min/15000 cells)
NT siRNA
UCP2 siRNA
***
***
*
Basal
Oligo-sensitive
Non-glycolytic

Figure S4 continued…

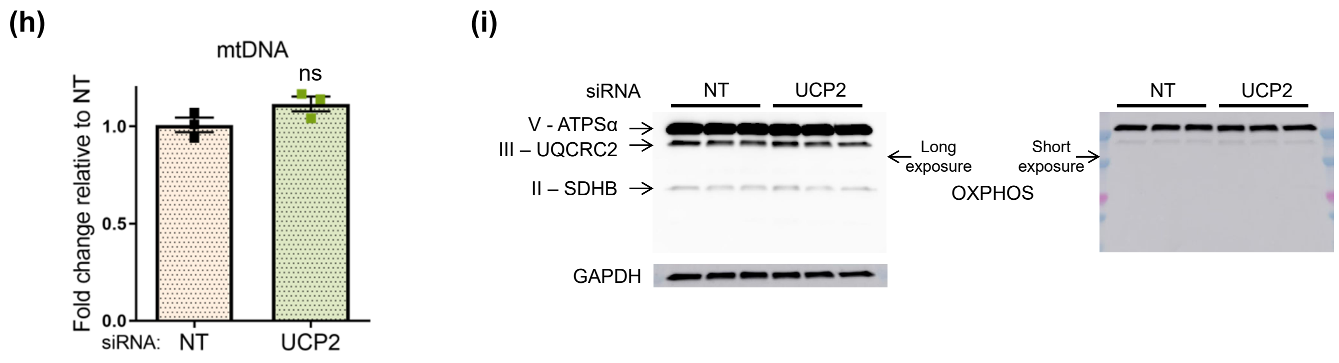

Figure S4. Mitochondrial Stress Test in IPF fibroblasts subjected to UCP2 silencing.

(**a, b**) IPF fibroblasts were subjected to siRNA-mediated silencing of UCP2 for a total of 72 hours. The fibroblasts were seeded 15,000 cells per well prior to measuring oxygen consumption rate (OCR) in an XFe96 analyzer (Seahorse Bioscience); graphical output depicted in (**a**); all individual components of OCR represented in (**b**). Error bars represent mean ± SEM (n=8); *p<0.05, **p<0.01. (**c**) IPF fibroblasts were subjected to siRNA-mediated silencing of UCP2 for a total of 72 hours, seeded at 15,000 cells per well, incubated with a substrate-restricted medium and treated with etomoxir 4 μM or vehicle at the indicated time point. OCR was measured; graphical output depicted. (**d**) IPF fibroblasts were subjected to siRNA-mediated silencing of UCP2 for a total of 72 hours, seeded at 15,000 cells per well, incubated with a substrate-restricted medium and then treated with bovine serum albumin (BSA) or BSA-Palmitate conjugate as per kit manufacturer's instructions, prior to measuring OCR; graphical output depicted. (**e**) IPF fibroblasts were subjected to siRNA-mediated silencing of UCP2, CPT1a or both for a total of 72 hours. The fibroblasts were seeded 15,000 cells per well and incubated in a substrate restricted medium prior to measuring OCR; graphical output depicted. (**f, g**) IPF fibroblasts with similar experimental conditions as in (A-B) were assessed for extracellular acidification rate (ECAR) in an XFe96 analyzer; graphical output depicted in (**f**); all individual components of ECAR represented in (**g**). Error bars represent mean ± SEM (n=8); *p<0.05, ***p<0.001. In all the studies above, blue arrows indicate the time points when etomoxir (*Eto*), oligomycin (*O*), Carbonyl cyanide-4-(trifluoromethoxy)phenylhydrazone (FCCP) (*F*), antimycin A/Rotenone (*A/R*) and 2-deoxy glucose (*2-DG*) were added to the cells as part of the Seahorse Mito Stress Test. (**h**) IPF fibroblasts were subjected to siRNA-mediated silencing of UCP2 for 72 hours. Mitochondrial DNA (mtDNA) content was assessed; graph shows relative expression compared to average of mtDNA content in fibroblasts treated with NT siRNA; mean ± SEM (n=3); difference statistically not significant. (**i**) IPF fibroblasts were subjected to siRNA-mediated silencing of UCP2 for 72 hours. Western blotting was performed to assess steady-state levels of proteins involved in mitochondrial oxidative phosphorylation – complex V (ATP synthase α; ATPSα), complex III (ubiquinol-cytochrome c reductase core protein 2; UQCRC2) and complex II (succinate dehydrogenase B; SDHB), using the Total OXPHOS Human WB Antibody Cocktail from Abcam. NT = non-targeting.

Figure S5

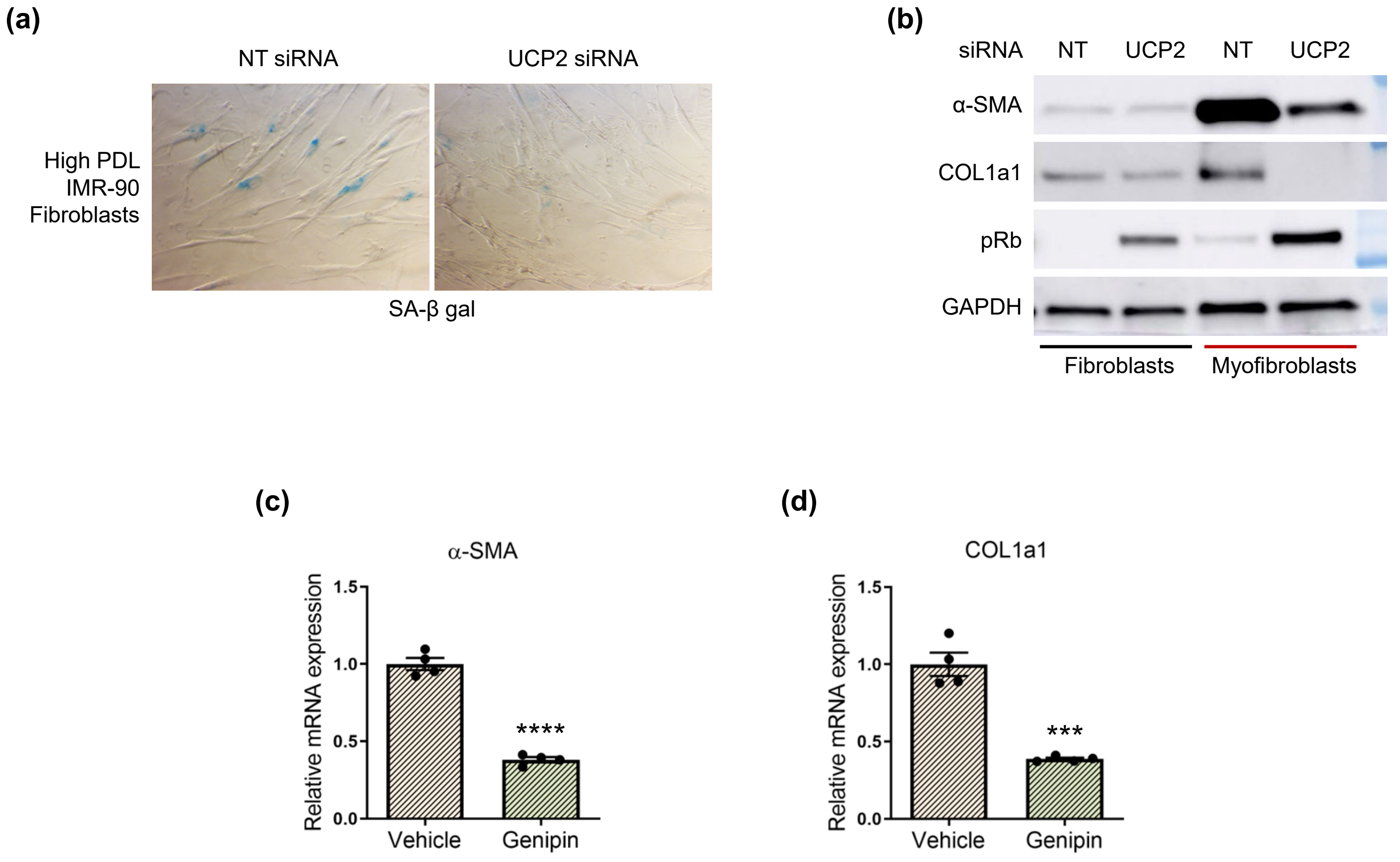

Figure S5. Effects of targeting UCP2 on IPF fibroblast senescence and myofibroblast differentiation.

(**a**) SA-β-gal staining was performed on replicatively senescent (high population doubling length – PDL >40) IMR-90 fibroblasts subjected to siRNA-mediated silencing of UCP2 for 72 hours; representative light microscopy images (10X) shown. (**b**) Fibroblasts derived from two different IPF lung explants, one with low and the other with high expression of myofibroblast markers α-smooth muscle actin (α-SMA) and collagen 1a1 (COL1a1), were treated with UCP2- (or non-targeting, NT-) siRNA for 72 hours. Western blotting was performed to assess steady-state levels of myofibroblast markers (α-SMA and COL1a1) and proliferation marker pRb. (**c, d**) IPF fibroblasts were treated with UCP2 inhibitor genipin 250 μM for 48 hours. Gene expression of myofibroblast markers α-smooth muscle actin (α-SMA) (**c**) and collagen 1a1 (COL1a1) (**d**) were assessed by qPCR. Graphs represent mean ± SEM (n=4 each group); ***p<0.001, ****p<0.0001.

Figure S6

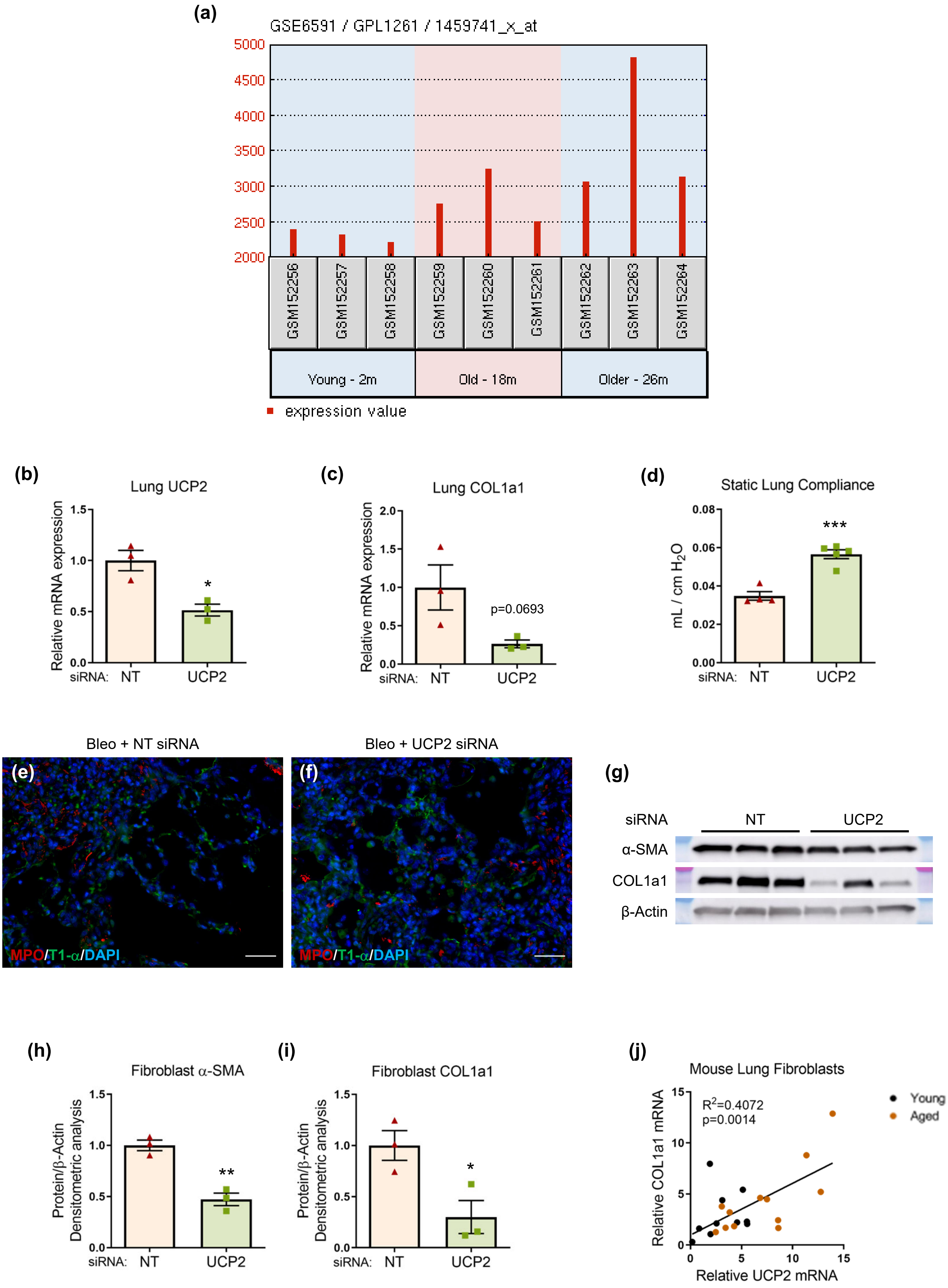

(a)
GSE6591 / GPL1261 / 1459741_x_at
5000
4500
4000
3500
3000
2500
2000
GSM152256
GSM152257
GSM152258
GSM152259
GSM152260
GSM152261
GSM152262
GSM152263
GSM152264
Young - 2m
Old - 18m
Older - 26m
expression value
(b)
Lung UCP2
Relative mRNA expression
siRNA: NT UCP2
(c)
Lung COL1a1
p=0.0693
(d)
Static Lung Compliance
mL / cm H2O
***
(e)
(f)
Bleo + NT siRNA
Bleo + UCP2 siRNA
MPO/T1-α/DAPI
(g)
siRNA NT UCP2
α-SMA
COL1a1
β-Actin
(h)
Fibroblast α-SMA
Protein/β-Actin Densitometric analysis
**
(i)
Fibroblast COL1a1
*
(j)
Mouse Lung Fibroblasts
R2=0.4072
p=0.0014
Young
Aged
Relative COL1a1 mRNA
Relative UCP2 mRNA

Figure S6. UCP2 expression lungs and lung fibroblasts of aged mice and therapeutic targeting of UCP2 in aged mice subjected to bleomycin injury.

(**a**) A publicly available GEO dataset, GSE6591 (https://www.ncbi.nlm.nih.gov/geo/query/acc.cgi?acc=GSE6591), was queried for UCP2 gene expression in the lungs among various age groups in C57BL/6J mice using the GEO2R tool; graphical output shown. (**b-g**) In the experiment described in **Figure 6b**, whole lung lysates (from the aged mice subjected to bleomycin injury and then treated with UCP2- or non-targeting siRNA) were assessed for gene expression of UCP2 (**b**) and collagen 1a1 (**c**) by qPCR. Graphs represent mean ± SEM (n=3 each group); *p<0.05. (**d**) Lung function was assessed using the flexiVent^{TM} apparatus; static compliance measured; graph represents mean ± SEM (n=4-5 each group); ***p<0.001. (**e, f**) Representative images show the areas of fibrotic remodeling and fluorescence patterns of myeloperoxidase (MPO, red), type 1 epithelial cells (T1-α, green) and nuclei (DAPI-blue) in indicated groups of mice. Scale bars = 50 μm. (**g**) Fibroblasts were isolated from the lungs and western blotting was performed to assess the steady state levels of myofibroblast markers α-smooth muscle actin (α-SMA) and collagen 1a1 (COL1a1); densitometric analyses shown in (**h**) and (**i**) respectively; graphs represent mean ± SEM (n=3); *p<0.05, **p<0.01. (**j**) In a separate experiment, young (2 months) and aged (18 months old) C57BL/6 mice were subjected to lung injury by instillation of oropharyngeal bleomycin (1.5 U/kg) (or no injury by instillation of PBS control). Lungs were harvested starting at 3 weeks up to 2 months after bleomycin injury; fibroblasts were isolated from the lungs and assessed for gene expression of UCP2 and collagen 1a1 (COL1a1). The graph represents correlation between mRNA expression of UCP2 and COL1a1.

Graphical Abstract

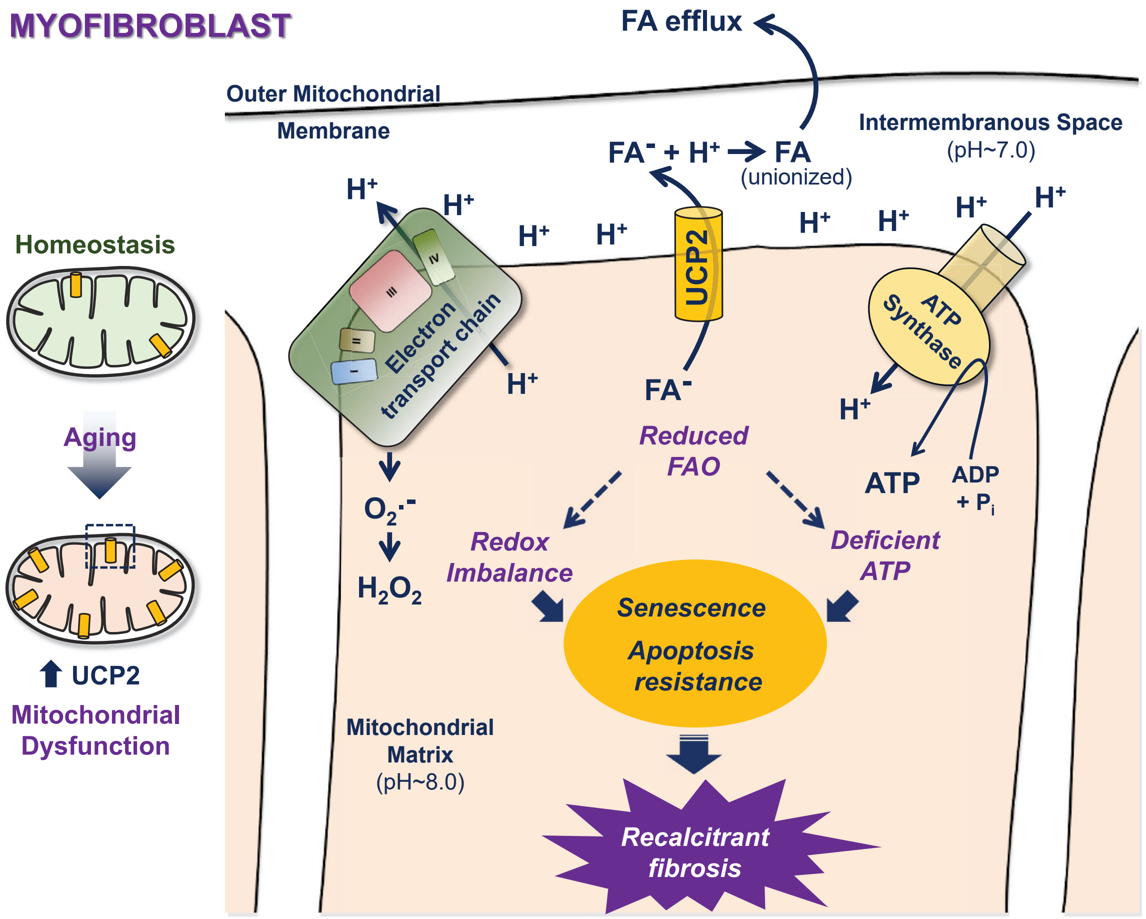

High constitutive levels of uncoupling protein-2 (UCP2) are observed with aging and in the age-related fibrotic disease, idiopathic pulmonary fibrosis (IPF). UCP2 functions as an exporter of fatty acid (FA) anions, which may account for the observed decrease in mitochondrial membrane potential of IPF myofibroblasts. Decreased coupling efficiency and fatty acid oxidation (FAO) lower ATP synthetic capacity in association with increased reactive oxygen species (ROS) production. These alterations promote induction of senescence and apoptosis resistance of myofibroblasts, hallmarks of recalcitrant and progressive fibrosis as seen in IPF.

KEY RESOURCES TABLE

REAGENT or RESOURCE	SOURCE	IDENTIFIER
**Antibodies and Stains**		
UCP2 (immunohistochemistry)	Proteintech	11081
UCP2 (western blotting)	Santa Cruz	sc-6525
Cyclin D1	Santa Cruz	sc-8396
pRb	Cell Signaling	9308s
α-SMA	ARP	03-61001
COL1a1	Abcam	ab138492
GAPDH	Abcam	ab9445
Cleaved PARP	Cell signaling	9541
p16	Cell Signaling	80772S
Total OXPHOS Human WB Antibody Cocktail	Abcam	ab110411
β-Actin	Sigma	A5316
JC-1 Dye	Invitrogen	T3168
LipidTOX	Invitrogen	H34351
MitoSOX™	Invitrogen	M36008
MitoTracker™ Green	Invitrogen	M7514
DAPI	Thermo Scientific	9541s
Ki-67	BD Biosciences	550609
TUNEL	Roche	12156792910
MPO	Abcam	ab208670
T1-α (Mouse podoplanin)	R&D Systems	AF3244
**Chemicals, peptides, and recombinant proteins**		
DMEM/F-12	Gibco	11320033
Fetal Bovine Serum	Gibco	10437028
Pen Strep	Gibco	15140
Trypsin-EDTA (0.25%)	Gibco	25200
DPBS pH 7.4	Gibco	14190
OptiMEM medium	Gibco	11058021
Lipofectamine 2000	Thermofisher	11668019
Dimethyl Sulphoxide (DMSO) TC grade	Sigma Aldrich	D2650-100ML
Nitrocellulose blotting membrane 0.45 mm	GE Healthcare Amersham	10600002
HEPES	Gibco	15630080
TRITON X-100	Sigma	T9284
The Dako Envision Dual Link System	Agilent	K406311-2
DAB/H_2O_2 kit	Vector Laboratories	SK-4100
Tween-20	Sigma Aldrich	P1379-4L

Collagenase type I	Advanced Biometrix	5005-100ml
Isoflurane	Piramal	NDC 66794-017-25
SYBR Green power up	Applied Biosystems	A25742
Bleomycin	Meitheal	NDC 71288-106-10
5X siRNA Buffer	Horizon Discovery	B-002000-UB-100
Hematoxylin	Vector Laboratories	H-3401-500
Masson's trichrome staining	Sigma	HT15-1KT
TRIS BASE	Fisher	BP1521
EDTA	BP118500	Fisher
Halt™ Protease and Phosphatase Inhibitor	Thermo Scientific	78442
RIPA	Thermo Scientific	89901
Diethylenetriaminepentaacetic acid (DTPA)	Sigma Aldrich	D6518-5G
Sodium chloride (NaCl)	Fisher Scientific	BP358-212
Potassium phosphate dibasic (HK_2PO_4)	Fisher Scientific	BP363-500
Potassium phosphate monobasic (KH_2PO_4)	Sigma Aldrich	P-5379
Krebs-Henseleit buffer (KHB)	(Alfa Aesar, Hill)	J67820
mito-TEMPO-H	Enzo Life Sciences	ALX-430-171-M005
Genipin	Sigma	G4796
XF calibrant solution	Agilent Technologies	100840-000
XF DMEM medium	Agilent Technologies	103575-100
100 mM pyruvate solution	Agilent Technologies	103578-100
200 mM glutamine	Agilent Technologies	103579-100
D-Glucose	Agilent Technologies	103577-100
Oligomycin	Sigma	O4876
FCCP	Sigma	C2920
Rotenone	Sigma	R8875
Antimycin A	Sigma	A8674
2-deoxy-D-glucose	Fisher	AC111980050
Etomoxir	Cayman Chemicals	11969
**Critical commercial assays**		
Pierce BCA protein assay kit	Thermo Scientific	23225
RNeasy Mini Kit	QIAGEN	74104

iScript reverse transcription kit	Bio-Rad	1708841
ATP Assay Kit	Abcam	ab83355
Deproteinizing Sample Preparation Kit	Abcam	ab204708
Fluorometric hydroxyproline assay	QuickZyme Biosciences	QZBHYPRO1
Seahorse XFe96 Cell Culture Microplate	Agilent Technologies	101085-004
Seahorse XFe96 Extracellular FluxPak mini	Agilent Technologies	102601-100
Seahorse XF Palmitate-BSA FAO Substrate	Agilent Technologies	102720-100
**Experimental models: Cell lines**		
IMR-90	ATCC	ATCC® CCL-186™
**Experimental models: Organisms/Strains**		
Mouse: C57BL/6J	Jackson Laboratories	Cat# 000664
**Oligonucleotides – primers**		
UCP2 (Forward): ACA AGA CCA TTG CCC GAG AG	This manuscript	N/A
UCP2 (Reverse): AGG AGG GCA TGA ACC CTT TG	This manuscript	N/A
α-SMA (Forward): TCC TCA TCC TCC CTT GAG AA	This manuscript	N/A
α-SMA (Reverse): ATG AAG GAT GGC TGG AAC AG	This manuscript	N/A
COL1A1 (Forward): ACG AAG ACA TCC CAC CAA TCA CCT	This manuscript	N/A
COL1A1 (Reverse): AGA TCA CGT CAT CGC ACA ACA CCT	This manuscript	N/A
GAPDH (Forward): ATC ATC CCT GCC TCT ACT GG	This manuscript	N/A
GAPDH (Reverse): CTG CTT CAC CAC CTT CTT GA	This manuscript	N/A
β-actin (Forward): TGC TAT CCA GGC TGT GCT AT	This manuscript	N/A
β-actin (Reverse): AGT CCA TCA CGA TGC CAG T	This manuscript	N/A
Mouse UCP2 (Forward): AAG TGT TTC GTC TCC CAG CC	This manuscript	N/A
Mouse UCP2 (Reverse): GGG ACC TTC AAT CGG CAA GA	This manuscript	N/A

Mouse GAPDH (Forward): GCA GTG GCA AAG TGG AGA TTG	This manuscript	N/A
Mouse GAPDH (Reverse): GGA GAT GAT GAC CCT TTT GGC TCC	This manuscript	N/A
Mouse α-SMA (Forward): GTC CCA GAC ATC AGG GAG TAA	This manuscript	N/A
Mouse α-SMA (Reverse): TCG GAT ACT TCA GCG TCA GGA	This manuscript	N/A
Mouse COL1A1 (Forward): GGA GGG CGA GTG CTG TGC TTT	This manuscript	N/A
Mouse COL1A1 (Reverse): GGG ACC AGG AGG ACC AGG AAG T	This manuscript	N/A
**Oligonucleotides - siRNAs**		
Human UCP2 siRNA (sense): AAU GCC UAC AAG ACC AUU G	This manuscript	N/A
Mouse/Rat UCP2 siRNA (sense): GAA GCC UAC AAG ACC AUU G	This manuscript	N/A
Human CPT1a siRNA (sense): GGG AGA GAA CCU CAU CAA UUU	This manuscript	N/A
Non-targeting siRNA (sense): UAA GGC UAU GAA GAG AUA C	This manuscript	N/A
**Software and algorithms**		
Prism 9.4	Graphpad software	https://graphpad.com
MetaboAnalyst 5.0	Open source software	http://metaboanalyst.ca
FlowJo	FlowJo software	https://www.flowjo.com/
Fiji	Open source software	https://imagej.net/Fiji
fibroXplorer	Open source software	https://www.fibroxplorer.com/perturbed-state-human
**Other**		
Keyence BZ-X700 microscope	Keyence	N/A
LSR-II flow cytometer	Becton Dickinson	N/A
XFe96 extracellular flux analyzer	Seahorse Biosciences	N/A
